# Metal Free Reversible-Deactivation Radical Polymerizations: Advances, Challenges, and Opportunities

**DOI:** 10.3390/polym10010035

**Published:** 2017-12-29

**Authors:** Johannes Kreutzer, Yusuf Yagci

**Affiliations:** 1 Department of Chemistry, Istanbul Technical University, Maslak, 34469 Istanbul, Turkey; 2 Center of Excellence for Advanced Materials Research (CEAMR) and Chemistry Department, Faculty of Science, King Abdulaziz University, P.O. Box 80203, Jeddah 21589, Saudi Arabia

**Keywords:** reversible-deactivation radical polymerization, NMRP, ATRP, RAFT, organic synthesis, polymer chemistry

## Abstract

A considerable amount of the worldwide industrial production of synthetic polymers is currently based on radical polymerization methods. The steadily increasing demand on high performance plastics and tailored polymers which serve specialized applications is driven by the development of new techniques to enable control of polymerization reactions on a molecular level. Contrary to conventional radical polymerization, reversible-deactivation radical polymerization (RDRP) techniques provide the possibility to prepare polymers with well-defined structures and functionalities. The review provides a comprehensive summary over the development of the three most important RDRP methods, which are nitroxide mediated radical polymerization, atom transfer radical polymerization and reversible addition fragmentation chain transfer polymerization. The focus thereby is set on the newest developments in transition metal free systems, which allow using these techniques for biological or biomedical applications. After each section selected examples from materials synthesis and application to biomedical materials are summarized.

## 1. Introduction

Conventional radical polymerization was already a widespread technique when Swarcz introduced the concept of living anionic polymerization for the first time in 1956 [1] and demonstrated its viability for the fabrication of block copolymers [2]. Differing from ionic polymerization, radical polymerization suffers from bimolecular termination reactions and it was therefore considered impossible to transfer the concept of living anionic polymerization to radical systems.

In radical polymerization, continuous generation of radical chains is necessary to establish a steady radical concentration which should be very low to reduce termination processes and to achieve high molecular weight polymers. Thus, initiation proceeds slowly and at any time during the polymerization reaction while the rate of propagation proceeds fast when compared to the rate of initiation. Reaction between the propagating polymer chain and a monomer occurs approximately every 1 ms and the lifetime of a propagating polymer chain, until it undergoes irreversible termination is approximately 1 s. This short reaction time essentially disables chemical functionalization or chain extension and makes the preparation of well-defined polymers or block polymers impossible.

**Scheme 1:**
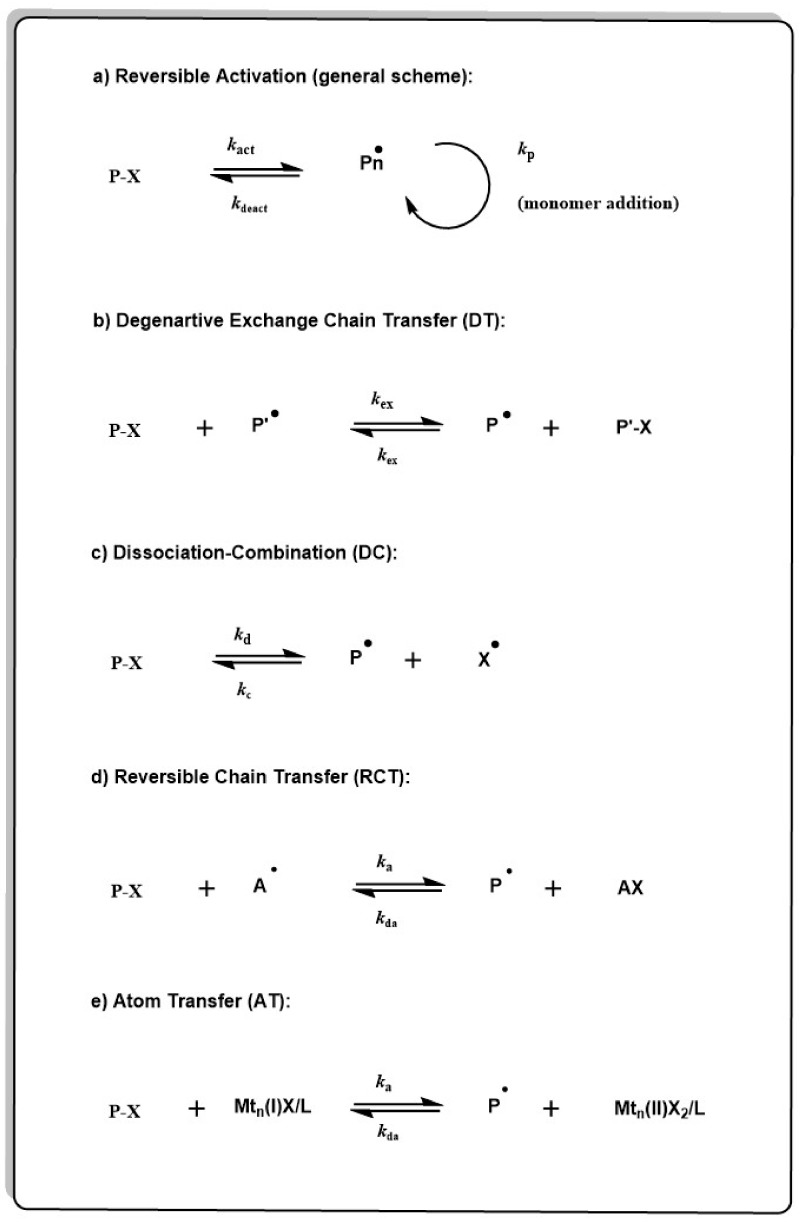
The most common mechanisms for reversible activation in polymerization reactions.

In reversible-deactivation radical polymerization (RDRP) different criteria must be met which allow the formation of polymers with narrow molecular weight distribution. Contrary to the conventional radical polymerization, initiation in RDRP must be fast to guarantee a uniform growth of the polymer chains starting from the beginning of the reaction. Long lifetime of growing chains allows chemical reactions, such as chain end-functionalization and chain extension. Furthermore, a low degree of termination reactions is necessary. Typically, these criteria are met by a dynamic equilibrium between a dormant and an active state which can be enabled by trapping radical chains with stable radicals or by reversible transfer processes. Different mechanisms for reversible activation-deactivation processes (see Scheme 1a) exist, leading to a manifold of different approaches to RDRP. In degenerative exchange chain transfer (DT, Scheme 1b) the dormant polymer is attracted by a propagating radical to form the active species and the dormant species, **P–X**. This mechanism can proceed in two ways: If the capping group is an atom or a simple group, it can be transferred from radical to radical without forming any kinetically important intermediate. Iodine mediated polymerization for example follows this mechanism. A second possibility opens up, if the capping group has a double bond which is accessible to the addition of a polymer. In this case the exchange reaction occurs with the addition of the polymer radical **P∙** to the polymer **P′–X** to form a **P^…^X^…^P′∙** intermediate, which can disproportionate to **P–X** and **P′∙**. Reversible addition fragmentation chain transfer polymerization (RAFT) is the most prominent example following this mechanism. In dissociation-combination (DC, see Scheme 1c) a capped polymer **P–X** dissociates in the individual radicals by thermal or photochemical cleavage. The capping radical must be stable and not undergo any other reactions except recombination with the polymer radical to give the dormant species again. A typical example for DC is nitroxide mediated radical polymerization (NMRP) where nitroxides act as a capping agent. Furthermore, two catalytic pathways exist to establish a dynamic equilibrium: In reversible chain transfer polymerization (RCTP, Scheme 1d) iodine complexes of Ge, Sn, N or P compounds (**AI**) mediate as catalysts the abstraction of capping iodine from dormant polymer species to form a complex **A^…^I**. Transfer of an iodine radical from this complex to the propagating polymer eventually leads to the formation of the dormant species **P–I** again. In atom transfer radical polymerization (ATRP, Scheme 1e), the **P–X** bond is activated by an activator complex (typically Cu or other transition metal (Mt_n_) complexes) or organic photo catalysts to release the polymer radical. Transfer of the halide group to the propagating polymer chain gives the dormant species again. 

DC, RCT and ATRP follow the persistent radical effect (PRE) which provides a self-regulating effect in RDRP. Persistent radicals, which are used as mediators in the reaction, cannot terminate with each other but only couple with the growing chain. Thus, every termination reaction between two propagating chains results in accumulation of persistent radicals, whose concentration steadily increases with time. This causes a decrease in the concentration of radicals and termination probability. Degenerative transfer is the system which shows the smallest perturbation of the free radical polymerization mechanism. Radicals are formed externally and polymerization stops when the source of radicals is consumed, polymerization proceeds under a steady state equilibrium as found in free radical polymerization. 

Degenerative transfer does not obey the PRE and follows typically free radical polymerization kinetics with slow initiation and fast termination. 

Termination in RDRP does occur, however, the risk is reduced by increasing the concentration of dormant species, establishing a fast exchange between active radicals and dormant species and reducing the polymerization rate. Self-termination can be quantified by determining the dead chain fraction (DCF) as given in Equation (1) and typically should not exceed 10% in RDRP [3].

(1) $$DCF=\frac{\left[T\right]}{\left[R-X\right]}=\frac{2DP_{T}k_{t}{\left[\ln(1-p)\right]}^{2}}{{\left[M\right]}_{0}k_{p}^{2}t}$$

DCF is defined as the ratio of the number of dead chains [T] to the number of all initiated chains [R − X]. Decreasing the degree of polymerization (*DP*_T_), conversion (*p*) or rate coefficients of termination (*k*_t_) to propagation (*k*_p_) as well as increasing monomer concentration [M]_0_ and reaction time (*t*) results in decreased DCF and increased chain end-functionality.

The lifetime of a growing chain is extended from ~1 s in free radical polymerization to 1 d in RDRP. Initiation happens fast and allows for nearly instantaneous growth of all chains. Furthermore, DCF in RDRP is below 10% while it is nearly 100% in free radical polymerization. Deactivation and activation reactions in RDRP, as well as the PRE in the case of NMRP and ATRP, allow the establishment of a steady radical concentration. In free radical polymerization and RAFT a steady radical concentration, however, is established by balancing the rate of initiation and termination. Termination reactions typically occur between newly generated short chains and long chains. In reactions following the PRE, initiation occurs fast at the beginning and all chains grow uniformly while the reaction proceeds. As a result, the probability of termination decreases with time.

Almost simultaneously with the emergence of RDRP, the concept of living polymers arose. Polymer chains formed by NMRP, ATRP or RAFT are end-functionalized and capable of initiating polymerization as macro initiators. Thus, synthesis of block copolymers became accessible. The synthesis of telechelic polymers by RDRP was summarized recently in a review article [4].

## 2. Nitroxide Mediated Radical Polymerization (NMRP)

NMRP became a widespread RDRP method during the last decades, particularly for styrene based monomers with various functionalities in the absence of expensive metal catalysts and under easily controllable reaction conditions. Polymers obtained by NMRP do not require additional purification steps.

### 2.1. Reaction Mechanism of NMRP

**Scheme 2:**
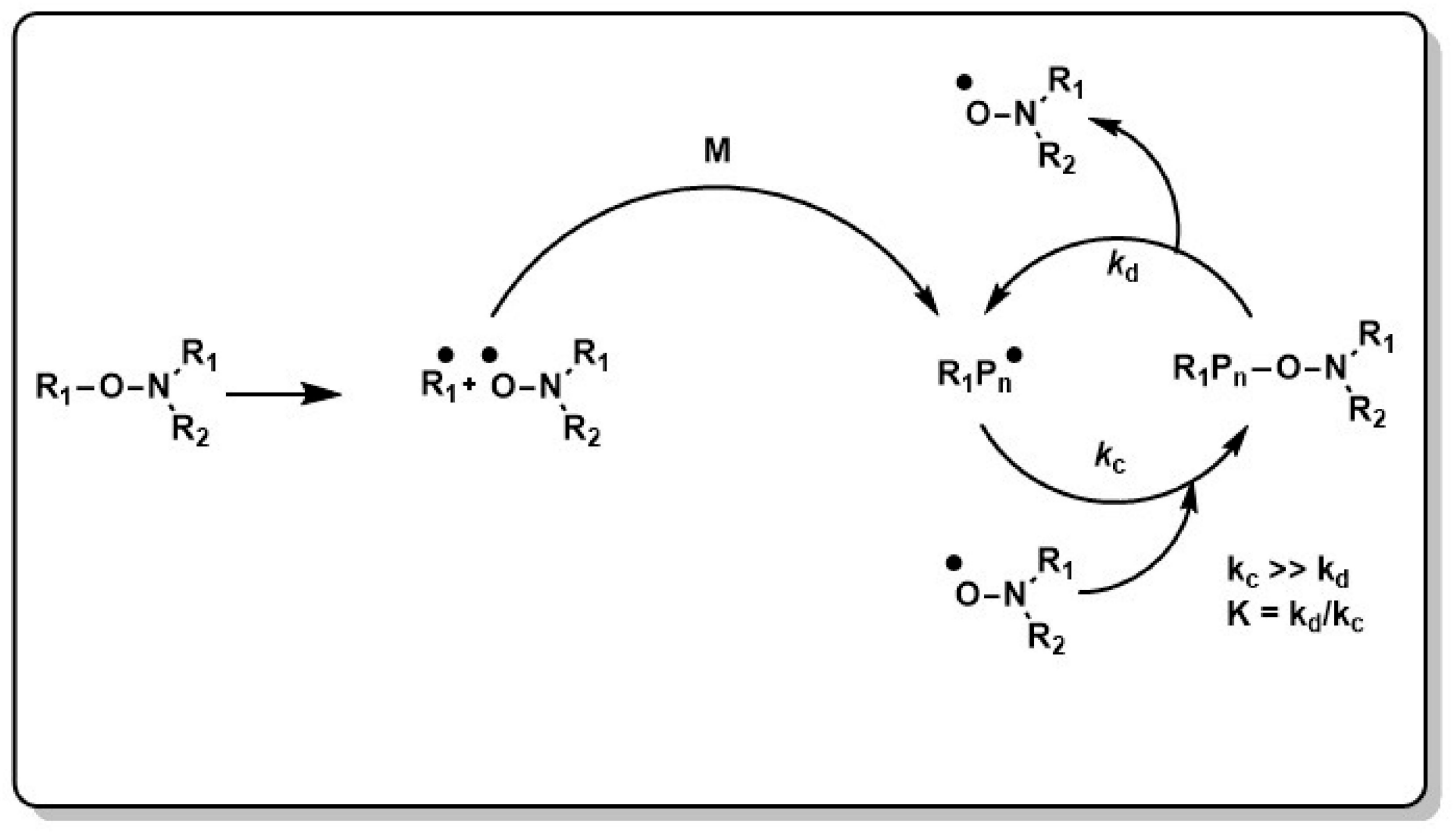
General scheme of the reaction mechanism in NMRP.

The control principle in NMRP is the reversible trapping of propagating polymer chains by stable nitroxide radicals and the formation of dormant species (see Scheme 2).

**Scheme 3:**
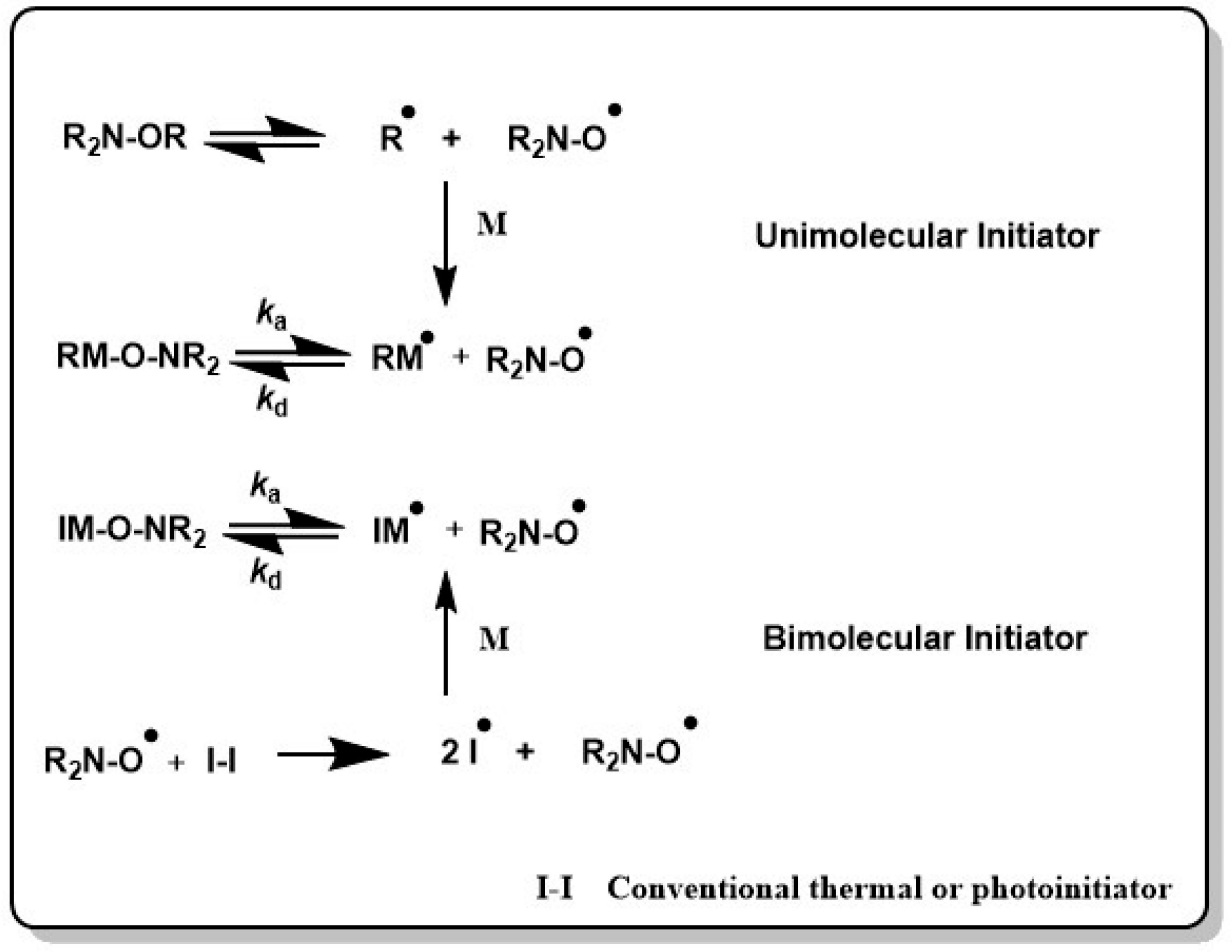
Initiation mechanism of uni- and bimolecular initiators in NMRP.

Shifting this equilibrium to the dormant form (*k*_c_ >> *k*_d_, e.g., excess of nitroxide) keeps the concentration of the active chains low, which minimizes irreversible chain termination. Initiation can be either performed in a bicomponent system, where a thermal initiator such as 2,2′-azobis(2-methylpropionitril) (AIBN) or benzoylperoxide (BPO) starts the polymerization reaction in the presence of a nitroxide radical, or in a unimolecular initiation process, where the nitroxide radical is generated through chemical, thermal or photochemical cleavage of an alkoxyamine (see Scheme 3). The nitroxide can then recombine with the propagating active chain to give a nitroxide terminated radical as dormant species. This macroalkoxyamine can undergo reversible C–ON bond homolysis at typically elevated reaction temperatures and releases the stable nitroxide radical as well as the active polymer chain, which continues to propagate. 

In the unimolecular approach, the concentration of the initiating radical, typically an alkyl radical which is formed after cleavage of the alkoxyamine, equals the nitroxide radical concentration which facilitates the control of the polymerization reaction. 

NMRP shows all typical features of a living polymerization reaction and tuning the alkyl as well as the nitroxide fragment in the unimolecular initiator allows for preparation of α- and ω-end-functionalized polymers. Extension of the alkoxyamine systems to bifunctional alkoxyamines allows for in situ “inside-out” or “outside-in” formation of block copolymers. Due to the living character of the reaction, block polymers are accessible and a vast amount of different complex polymer structures were lately prepared by NMRP.

### 2.2. Development of Nitroxides for NMRP

**Chart 1:**
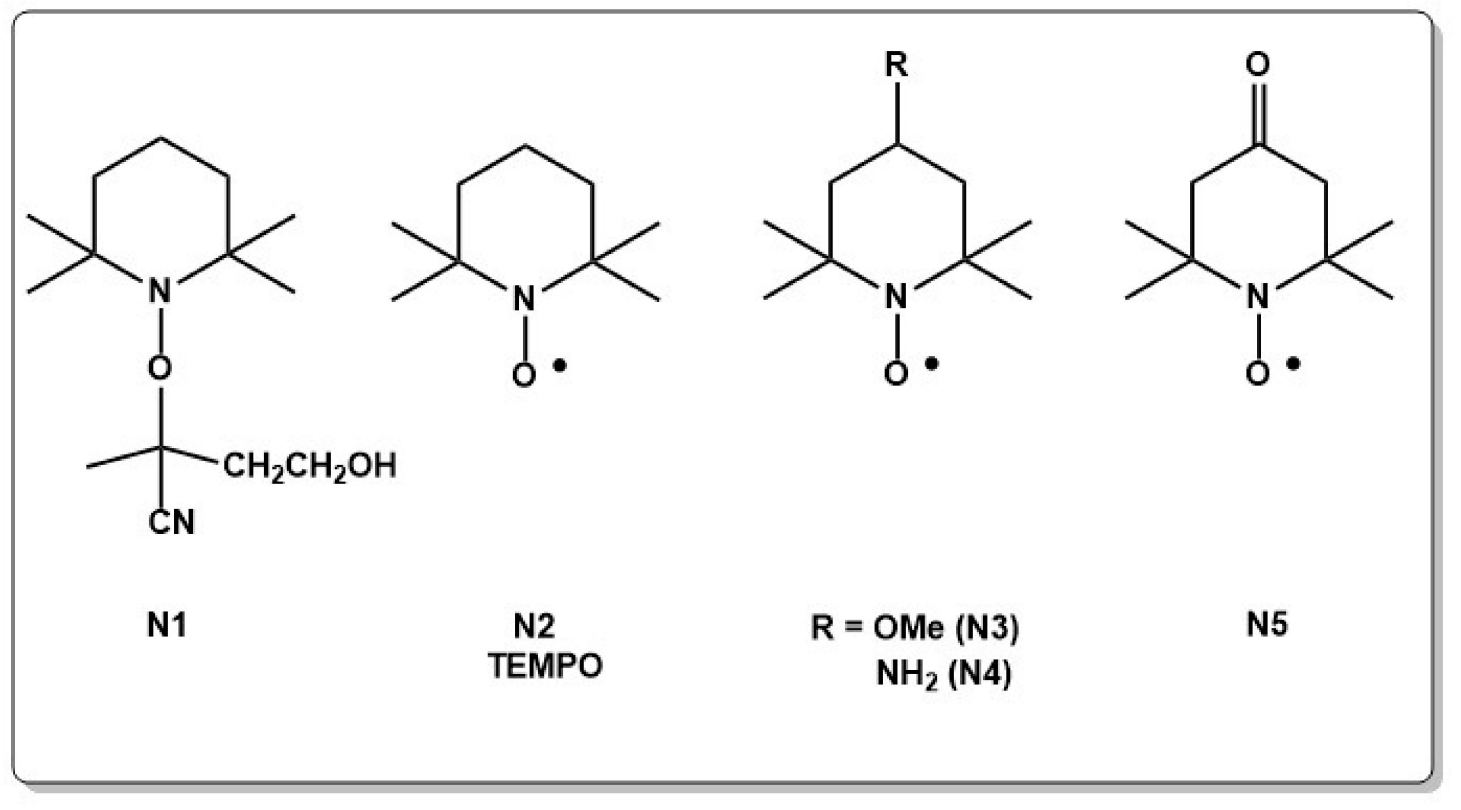
TEMPO derivatives used as nitroxides in NMRP.

The development of NMRP dates back to research efforts at CSIRO in the seventies to reveal the chemistry of initiating polymerization reactions by radical trapping methods using nitroxides [5]. At this time, it was well known that nitroxide radicals favorably scavenge carbon centered radicals at near diffusion controlled rates and at low temperatures e.g., 40–60 °C which are typical for free radical polymerization reactions [6,7,8,9,10,11,12]. Important results from the trapping experiments, which led to the development of NMRP later on, can be summarized as: (i) trapping experiments with some nitroxides resulted in the formation of unstable products [13,14], (ii) some nitroxides showed isomerization upon heating or ageing [15] and (iii) small amounts of oligomeric products were found [13,16,17,18]. From these observations it was concluded, that nitroxides are able to undergo reversible dissociation under free radical polymerization conditions, a prerequisite for a RDRP approach. Based on the work of radical trapping experiments, the first successful NMRP polymerization was carried out in 1982 by thermally activating the trapping/dissociation of 1-(1-cyano-1-methylethoxy)-2,2,5,5-tetramethylpyrrolidine (**N1** in Chart 1) as the nitroxide in the presence of methyl acrylate (MA) at 80 °C. In this attempt, shorter MA oligomers which were end-functionalized with the nitroxide fragment, could be isolated [19]. Further experiments to improve the technique were then carried out based on a bi-component system using a thermal initiator such as AIBN or BPO in combination with a wide range of nitroxides [20]. It soon became clear that the major drawback of this system is the poor reproducibility due to primary radical initiation reactions which are hard to control. Therefore, a new concept was introduced by Rize and Hawker [19,21,22,23,24], who used so-called unimolecular initiators. Upon heating, such unimolecular initiators decompose to form alkyl and nitroxyl radical fragments. The unimolecular approach led not only to a better control of the polymerization due to an exact stoichiometric ratio of initiating alkyl radical and mediating nitroxide radical but pathed also the way to α- and ω-end-functionalized polymers as will be outlined below [21]. Hawker elegantly showed with labelling experiments that the nitroxide moiety does not necessarily stay on one particular polymer chain end but migrates from one chain to another during the polymerization reaction [25]. During the last decades, development of new initiating systems [26,27], improvement of nitroxides [20,28,29,30,31], accelerating the polymerization rates by using additives [32,33,34,35,36,37] or additionally added initiators [38,39,40] and the possibility to synthesize complex polymer structures [41,42,43,44,45,46,47,48,49,50,51,52,53,54,55,56,57] in combination with the easiness of the method, which does not need high purity chemicals or water free systems further subsidized the success of NMRP. While first experiments which were carried out with 2,2,6,6,-tetramethylpiperidin-1-oxyl (TEMPO) (**N2** in Chart 1) as the nitroxide and styrene (St) as the monomer were successful, it became soon evident that tuning the electronic structure and the steric demand of the nitroxide is a crucial point in further developing NMRP [28,58,59,60,61,62]. TEMPO was mainly limited to polymerization of St at high temperatures and long reaction times [20]. Thus, a demand in improving the performance of nitroxides in NMRP led to increased efforts to understand the electronic and steric effects of nitroxides and alkoxyamines. First attempts made use of the molecular structure of TEMPO and soon derivatives with substituents in *para* position (**N3**–**N5** in Chart 1) were developed. Changing the substituent in *para* position allowed not only for the introduction of functional groups but also showed influence on the reactivity and 4-oxo TEMPO (**N5** in Chart 1) rapidly became the first nitroxide which allowed polymerization of a wide range of monomers [63]. 

**Chart 2:**
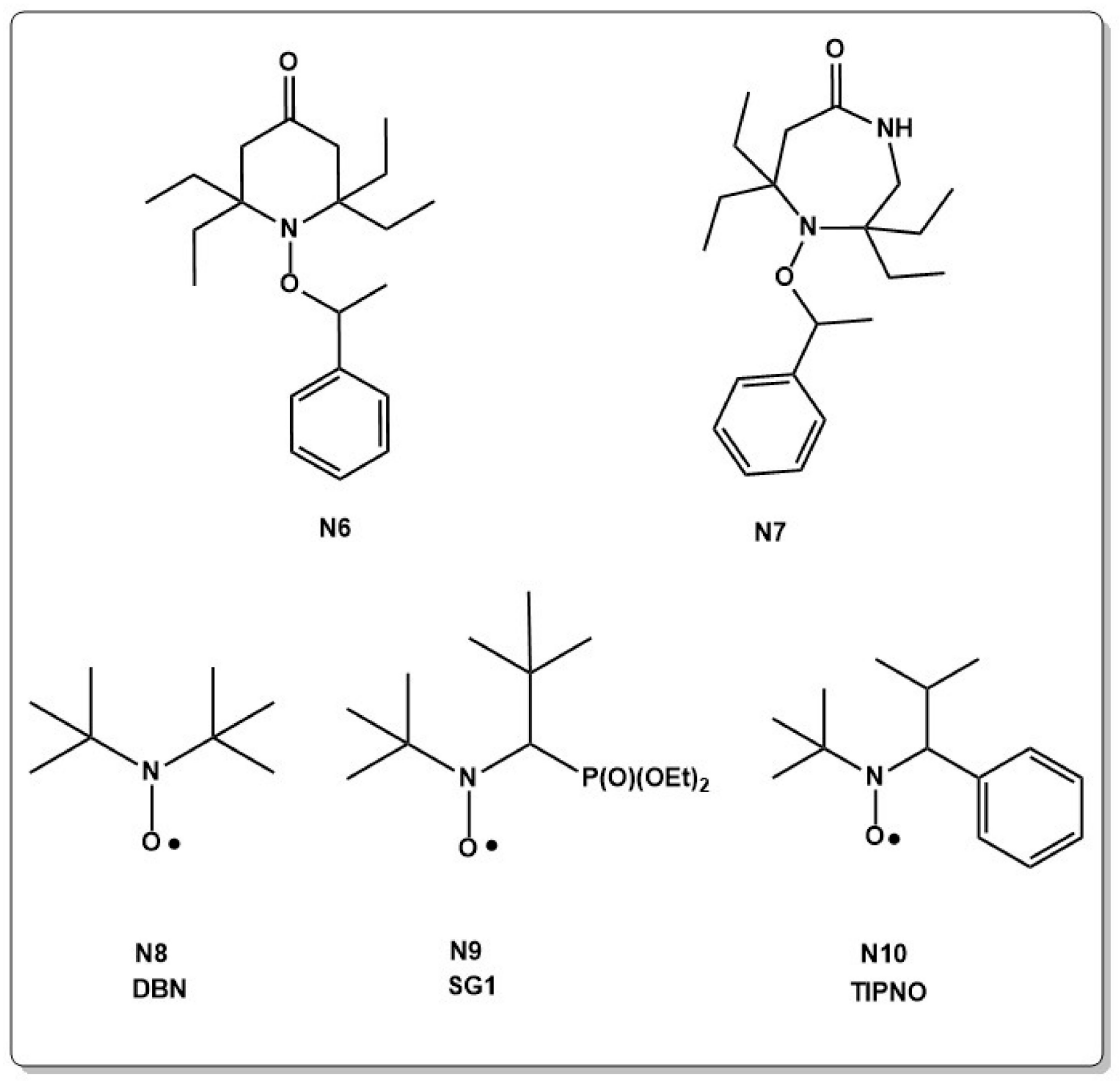
Alkoxyamines and nitroxide derivatives showing improved bond hydrolysis. Ring enlargement in TEMPO derivatives (**N6** and **N7**) and acyclic nitroxides (**N8**–**N10**).

High polymerization rates and molecular weight distribution rely on facile bond homolysis of the alkoxyamine [58]. However, if the C–ON bond is too labile, the reaction does not proceed in a controlled fashion. Strong bonds on the other side can inhibit the polymerization. Thus, a lot of work was devoted to adjusting the bond homolysis behavior of different alkoxyamines. Experimental and theoretical work showed that the stabilization of the radical after homolysis, or the destabilization of the alkoxyamine before homolysis facilitates a bond homolysis [58,59,64,65,66,67,68,69,70,71,72,73,74,75,76,77]. Thereby, steric effects have a more important influence on the bond dissociation energy than electronic effects [58,78,79,80]. Spiro type alkoxyamines are, for example, sterically crowded close to the C–ON bond and allow for polymerization at temperatures as low as 50 °C. Ring enlargement of the well investigated 2,2,6,6-tetraethyl-1-(1-phenylethoxy) piperidin-4-one (**N6** in Chart 2) to the seven membered alkoxamine 2,2,7,7-tetraethyl-1-(1-phenylethoxy)-1,4-diazepan-5-one (**N7** in Chart 2) on the other side resulted in a three times higher equilibrium constant and increased conversion at lower temperatures [58,59,60,81]. 

Overcrowding, however, can introduce negative effects on the polymerization control. In 2006 Studer and co-workers started an attempt to investigate in detail the steric effects and to estimate a possible maximum in steric crowding at TEMPO based alkoxyamines. The results made clear that steric influence mainly effects the recombination between the nitroxide radical and the living chain end. Very bulky substituents decrease the possibility of a trapping reaction, which results in loss of control during the polymerization [82]. 

NO–C vs. N–OC bond cleavage was theoretically investigated on a large set of alkoxyamines and showed that the free energies of the NO–C homolysis depends on the properties of the alkyl fragment, while the free energies of the N–OC bond homolysis depends more on the properties of the nitroxide fragment. N–OC bond homolysis is only favored when heteroatoms can be found in α-position of the NO–C carbon. Furthermore, acyclic and indoline type alkoxyamines have a higher tendency to C–ON homolysis than other cyclic alkoxyamines [83]. A big step forward in alkoxyamine design was therefore the development of acyclic nitroxides such as the nowadays commercially available di-*t*-butyl nitroxide (DBN, **N8** in Chart 2). Acyclic alkoxyamines showed to have much higher dissociation constants than the cyclic nitroxides. It was therefore not surprising that the breakthrough in the development of nitroxides was the design of acyclic alkoxyamines, such as *N*-*t*-butyl-1-diethoxyphosphoryl-*N*-oxidanyl-2,2-dimethylpropan-1-amine (SG1, **N9** in Chart 2) and 2,2,5-trimethyl-4-phenyl-3-azahexane-3-nitroxide (TIPNO, **N10** in Chart 2). The latter, which was developed by the groups of Tordo [61] and Hawker [28], was the first nitroxide that effectively polymerized a wide range of monomers in a controlled fashion. SG1 and TIPNO are still one of the most effective and widely used nitroxides so far [62]. 

**Chart 3:**
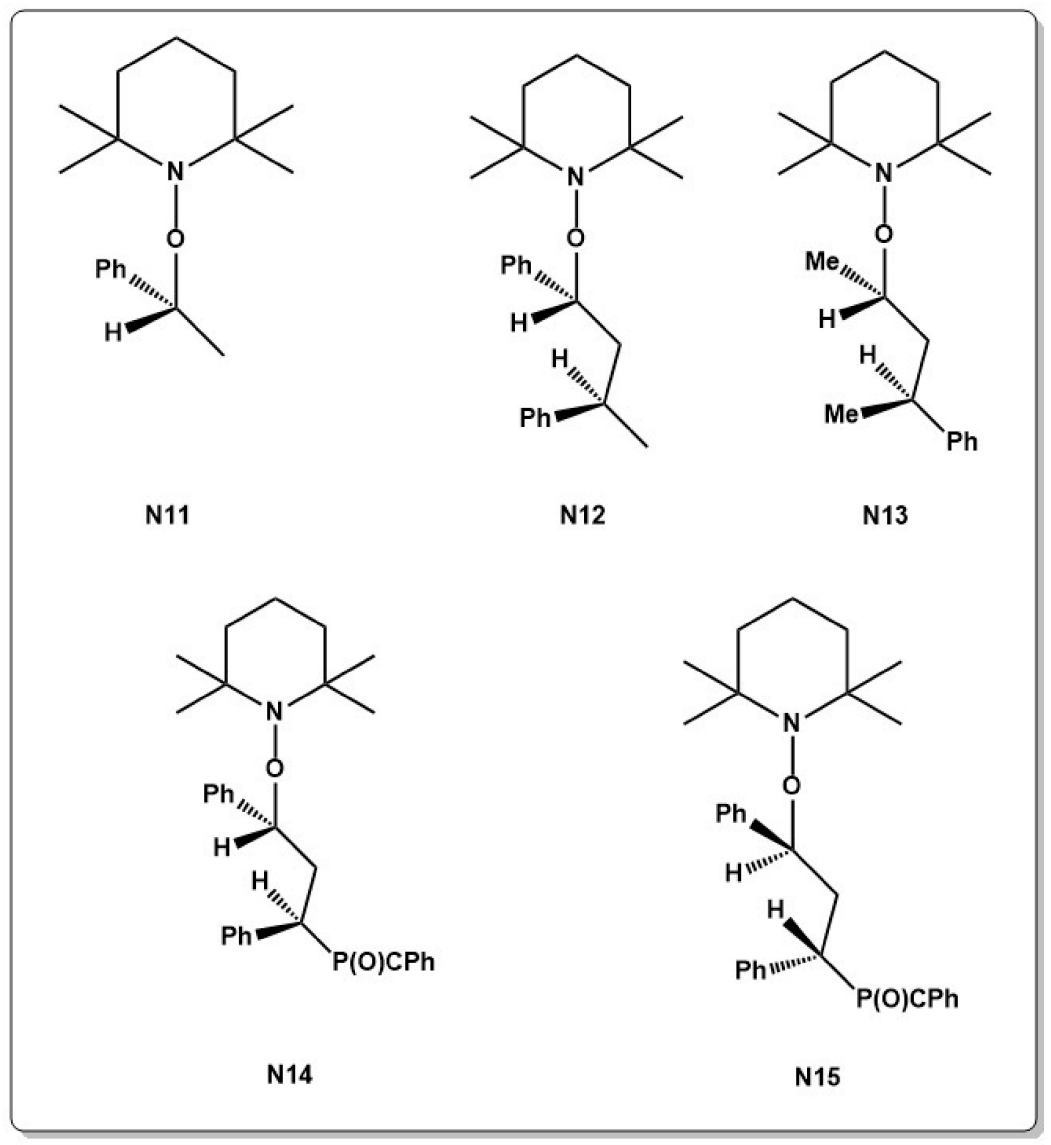
Alkoxyamines bearing stereo centers used in NMRP.

Diastereomeric effects on the homolysis of C–ON bond were observed in a theoretical study by Marque and co-workers in TEMPO alkoxyamine systems, bearing stereo centers on the alkoxy fragment (**N11**–**N15** in Chart 3). The difference in the dissociation constant was identified as an entropic effect of the transition state of the bond dissociation. For the *RS*/*SR* pair the released alkyl radical showed the same conformation as in the alkoxyamine while for the *RR* and *SS* pairs, this effect was not observed. Thus, reorganization had to occur for the *SS*/*RR* pairs during bond cleavage, which increases the entropy term [84]. 

Challenges in the reaction control of NMRP concern typical side reactions such as bimolecular self-termination of transient polymeric radicals, competing N–OR bond homolysis, formation of mid-chain radicals, chain transfer to the solvent and disproportionation, alternatively named H-transfer or cross-termination. While most of the side reactions can be minimized by adjusting the reaction conditions and the choice of the alkoxyamine, cross-termination remains a bigger drawback, especially for monomers such as MA. In cross-termination, a proton is abstracted from the propagating chain end to give an olefin terminated polymer chain and a hydroxylamine. This results in the formation of dead chain ends and loss of control over the polymerization reaction with increased dispersity (D) [85,86,87]. Double bonds on the polymer chain end suffer from auto oxidation and decrease the service life and performance of the polymer [88]. The hydroxylamine can transfer the proton to another living polymer chain, thus giving a second dead end [89].

**Chart 4:**
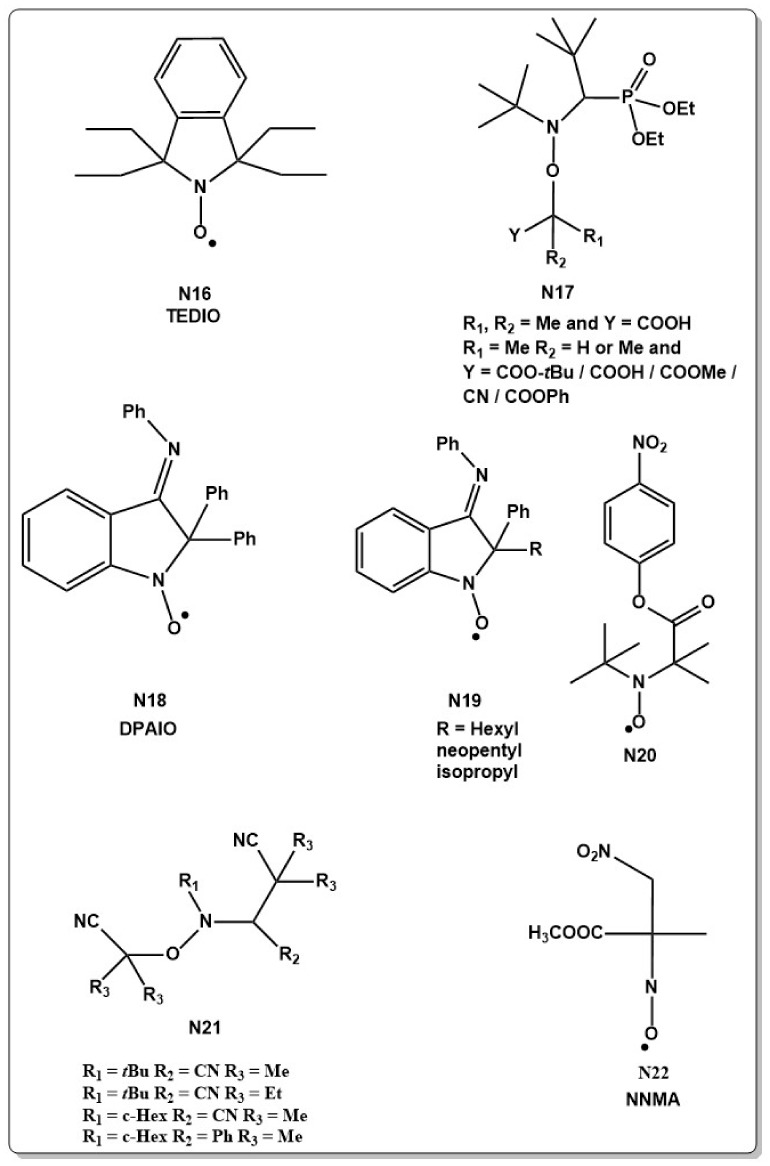
Nitroxides and alkoxyamines used for the RDRP of MMA in NMRP.

This disproportionation reaction becomes especially evident in the polymerization of methyl methacrylate (MMA) [86,90,91,92,93] which appeared to be an on-going challenge in NMRP. Since the first reports, NMRP was often criticized due to its inefficient polymerization of MMA which shows typically a low degree of control over polymerization. Poly(methyl methacrylate) (PMMA), however, is considered to be one of the most important industrial polymers. Thus, many studies were devoted to improving the controlled character. Different TEMPO and 1,1,3,3,-tetraethylisoindolin-2-oxyl (TEDIO, **N16** in Chart 4) based nitroxides were tested by Rizzardo but conversion remained relatively low in the range of 30%–40% [90]. 

The development of SG1 and TIPNO nitroxides could partially solve this problem, however the reaction equilibrium was then shifted to the active radical and an increase in termination reactions and low conversion rates were observed [94,95]. Copolymerization of MMA with small amounts of St or other co-monomers shifts the equilibrium to the dormant species and allows reduction of termination reactions [96,97]. Presence of 4%–8% St at 90 °C for example allowed to polymerize MMA in a moderately controlled manner [96,98]. Different tertiary SG1 type alkoxyamines (**N17** in Chart 4) were developed, which allowed to decrease the reaction temperature further to below 50 °C, making the polymerization reaction less prone to side reactions at lower temperatures [99] but the SG1 system did not show to be very suitable to polymerize MMA. A large dissociation constant and a small recombination constant indicates that the equilibrium constant becomes too large to effectively control the polymerization [94]. Although polymerization reactions with MMA at low temperatures were carried out successfully, a small amount of co-monomer was always necessary to prevent side reactions. 

Besides St, acrylonitrile (AN) [100,101], 9-(4-vinylbenzyl)-9H-carbazole (4VBC) [102,103,104], or cyclic ketene acetals [105,106] were used as a co-monomer to control the polymerization of MA. However, copolymer formation is not always desired, even if the amount of co-monomer is small. For this reason, development of NMRP systems that allow the RDRP of MMA in the absence of co-monomer was long sought after. The requirements for successful NMRP of MMA are a low reactivity, which prevents side reactions such as disproportionation, slow homolysis and fast recombination ability [94,107,108]. Indol type nitroxides fulfill part of these conditions and 2,2-diphenyl-3-phenylimino-2,3-dihydroindol-1-yloxyl (DPAIO, **N18** in Chart 4), was used to homopolymerize MMA for the first time with 80% conversion in a controlled way. The chain-end fidelity was investigated by chain extension experiments [108]. Based on this work, new DPAIO derivatives were developed for the polymerization of MMA at low temperatures (**N19** in Chart 4) [109,110,111] but showed in general a high (D), indicating that efficient control was not achieved. Furthermore, experiments to polymerize other monomers than MMA with this class of nitroxides were not successful. Similarly, 4-nitrophenyl-2-methylpropionat-2-yl nitroxide (**N20** in Chart 4) showed control over MMA polymerization but failed to polymerize other monomers such as St [112]. Lately a new family of nitroxides (**N21** in Chart 4) was reported to be able of polymerizing MMA as well as St under good control and the livingness was shown by chain extension experiments [92]. A new approach involving *in-situ* NMRP at 50 °C to polymerize MMA was recently reported, giving MMA with narrow molecular weight distribution. Methyl-2-methyl-3-nitro-2-nitrosopropionate (NMMA, **N22** in Chart 4) was used as a mediator and the reaction was thermally initiated with the low temperature azo initiator 2,2′-azobis(4-methoxy-2,4-dimethyl valeronitrile) (V70) [113]. The polymerization showed a linear behavior in the time-conversion plot, which points to a RDRP. The observed relatively long induction time of approximately 2 h is considered to be typical for in-situ NMRP, as time is needed for the nitroxide to be formed. Furthermore, RDRP of benzyl methacrylate (BzMA) and trifluoroethyl methacrylate with the same nitroxide also appeared to be successful.

A relatively low polymerization rate independent of the employed monomer system is a further shortcoming of NMRP. Special accelerating agents such as organic acids [32,33,34], polar additives [35], reductants [36] or alkylating agents [37] could partially circumvent this problem. Lately, it was shown that also electron accepting monomers could improve polymerization rates. Copolymerization of St with acceptor monomers such as maleimide-*N*-phenylmaleimide (NPI), *N*-benzylmaleimide (BMI) and *N*-cyclohexylmaleimide (CMI) resulted in shortened induction periods and an increase of polymerization rates when compared to St homopolymerization [114]. Thereby, the polymerization rate increased by a factor of 5–8 in the sequence NPI > BMI > CMI.

**Chart 5:**
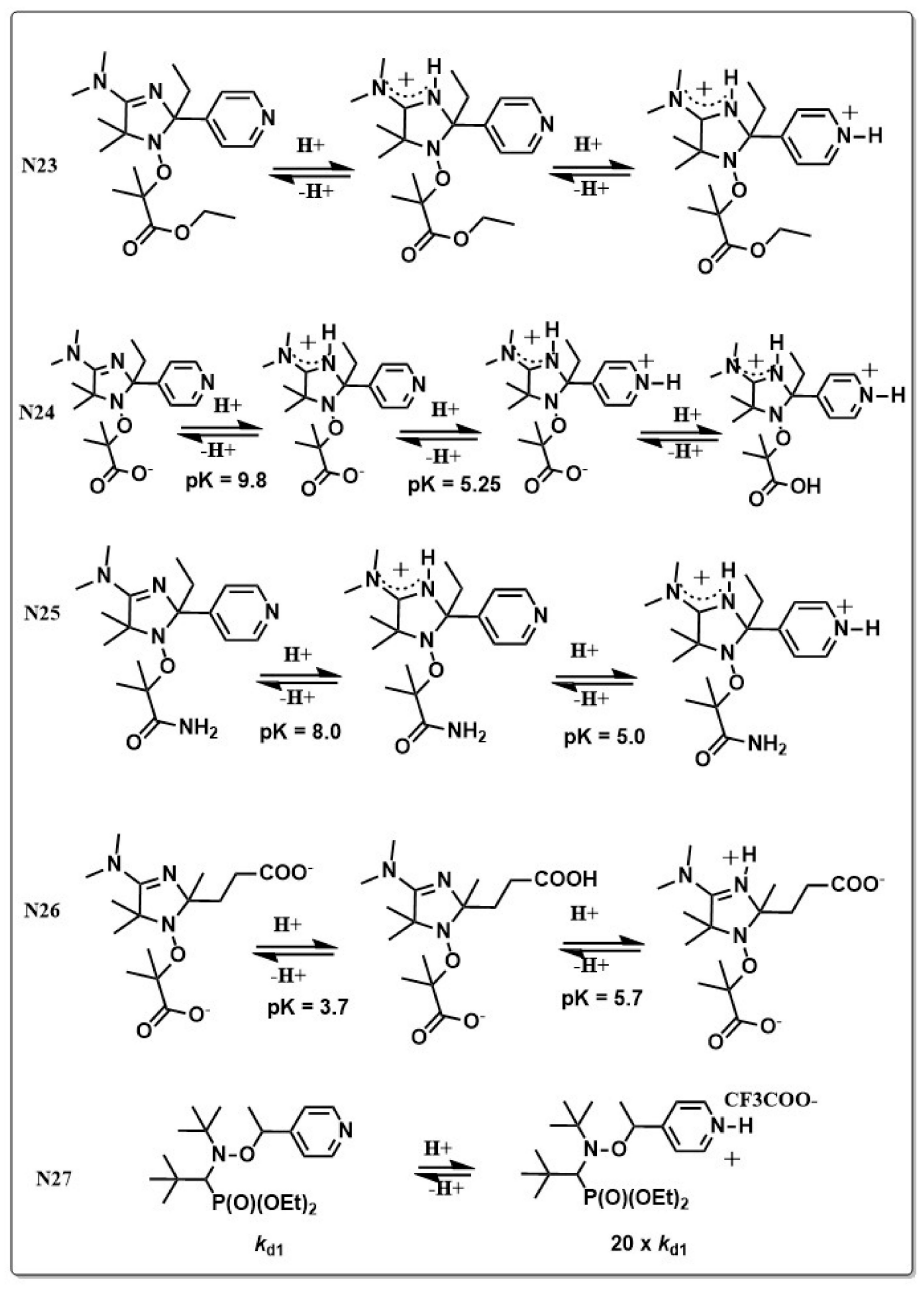
Alkoxyamines for pH triggered C–ON bond homolysis used in NMRP.

Most of the alkoxyamine initiator/mediator systems are based on thermal cleavage of the alkoxyamine. Lately much effort has been put into developing new initiating systems that can be either chemically or photochemically triggered. The first pH triggered C–ON bond homolysis and successful application in NMRP was reported in 2011 with imidazoline derivatized alkoxyamines [115]. Imidazole alkoxyamines (**N23**–**N26** in Chart 5) bearing different protonable groups were prepared and the polymerization of St, acrylamide (AAm) and styrene sulfonate (SS) in organic and aqueous media showed successful polymerization. The degree of control, as well as the molecular weight of the formed polymer were dependent on the pH. Both protonation of the alkoxide moiety [116] or the nitroxide moiety [115] can be used to trigger chemically C–ON homolysis. Protonation of the alkoxyamine results in a drastic change of the dissociation constant, which can be increased up to 64-fold compared to the unprotonated form. The increase of the dissociation constant might stem from a higher partial negative charge on the carbon of the C–ON bond and a stabilization of the alkyl radical [117]. Furthermore, the choice of counter ion had a drastic effect as well [118]. The dissociation constant in (**N27** in Chart 5) e.g., increased by 20 times when going from the unprotonated form to the protonated form with trifluorocarboxylate as counter anion. Changing the counter anion from triflate to camphorsulfonate showed an 80-fold increase when compared with the unprotonated form. This effect was explained by the solubility characteristics of the alkyl radical depending on the counter anion [118]. Furthermore, the counter anion of an activated species can help to stabilize the formed alkyl radical in solution after bond homolysis. Based on a lately published theoretical study, several TIPNO derivatives are able to control NMRP of St at room temperature when their acid groups are deprotonated, while the protonated form remains inactive and only controls polymerization at elevated temperatures. The reason was again found to be a stabilizing effect of the anion on the radical [119]. 

A completely different possibility to trigger NMRP was shown by photoacid generators. A tricomponent system composed of 4-methoxy TEMPO (MTEMPO) (**N3** in Chart 1) and bis(alkylphenyl)iodoniumhexafluorophosphate (BAI) as the photo-acid generator and AIBN as initiator were effective in RDRP of MMA at room temperature [26]. In this approach, the photosensitizer initiates the disproportionation of the AIBN initiator and the nitroxide mediates the reaction. Further studies of this system concentrated on the influence of different initiators [120,121] and on the polymerization of different monomers [27,122,123,124,125], on the performance of alkoxyamines instead of nitroxides as mediators [126] and on the study of different photoacid generators [121,127]. Eight different azo-initiators in combination with different photoacids were examined and control over the polymerization reaction could be achieved in all cases. Expectedly, photoacids with higher excitation coefficients showed a higher control of the polymerization and provided higher initiator efficiency. The half-life of the azoinitiators on the other side had little to no effect on the control or the molecular weight of the polymers thus formed. A detailed mechanistic study, however, is lacking until now. Isopropyl thioxanthone (ITX), BPO and 2,2-dimethoxy-2-phenylacetophenone (DMPA) were used in combination with TEMPO and SG1 alkoxyamines bearing an α-hydrogen at the C–ON carbon. All experiments were carried out with Ebercyl 605 resin and gave conversions of approximately 40% in a reasonable time. A mechanism was proposed, in which the photo-initiator in the excited state abstracts a proton from the alkoxyamine, leading to a carbon centered C–ON radical which facilitates C–ON bond homolysis [128,129]. 

**Chart 6:**
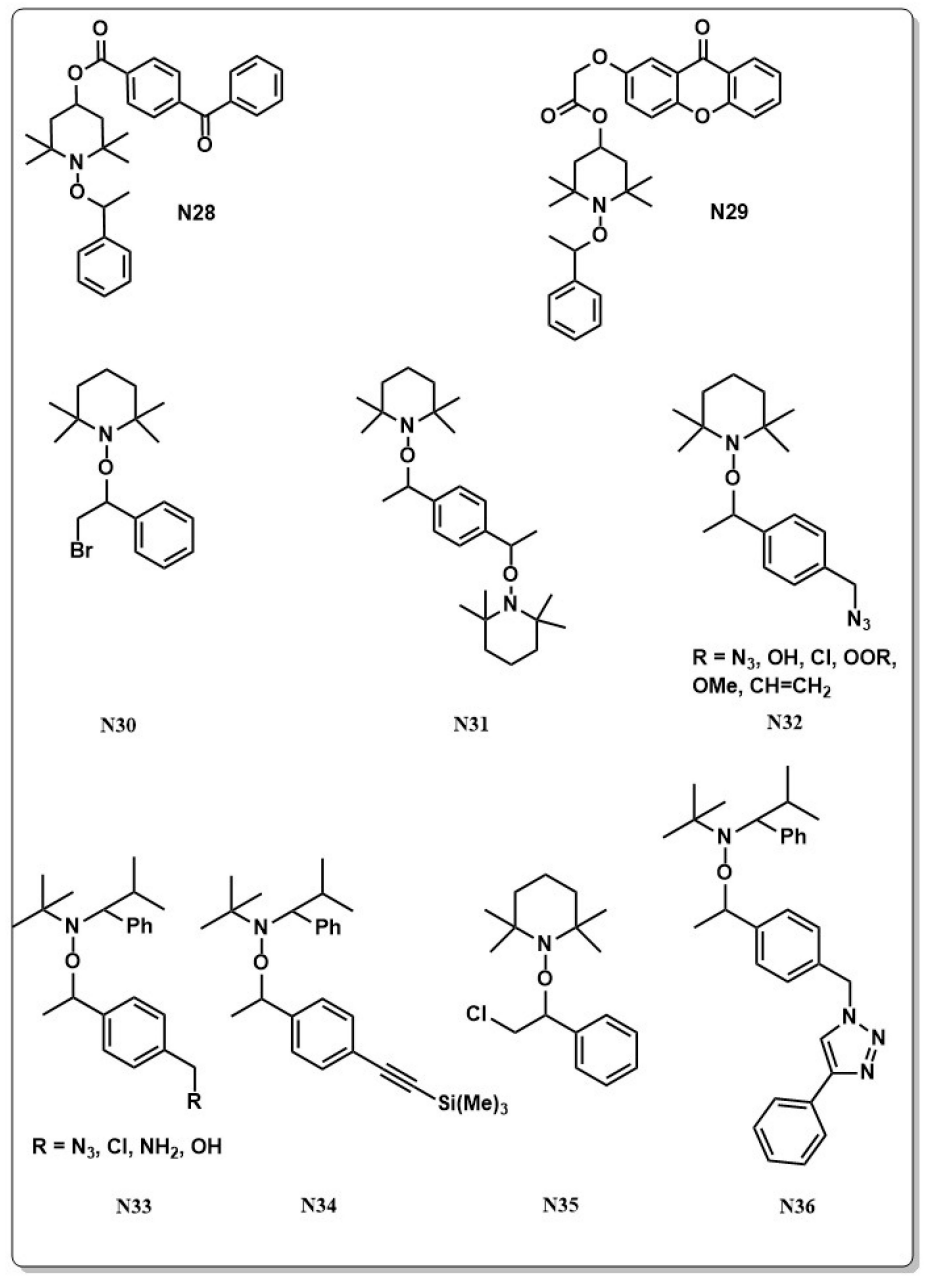
Functional alkoxyamines used in NMRP.

Direct attachment of chromophoric groups to the alkoxyamine guarantees a more efficient energy transfer and the design of so-called photoiniferter alkoxides (**N28**, **N29** in Chart 6) showed controlled characteristics during the polymerization. The first report on RDRP NMRP with photoiniferter alkoxyamines was published by Lalevee and Gigmes and co-workers in 2010, who attached a chromophore group directly on an acyclic alkoxyamine to yield the (methyl 2-((4-benzoylphenyl) ((1-methoxy-2-methyl-1-oxopropan-2-yl) oxy)-amino)-2-methylpropanoate iniferter (**N28** in Chart 6). Laser flash photolysis revealed that the major contribution to the C–ON bond homolysis stems from cleavage in the singlet state and polymerization of butyl acrylate (BA) with a partially living character was demonstrated [130]. Full control over the reaction was not achieved, mainly because of similar bond dissociation energies of the C–ON and C–NO bond. This problem, however, can be overcome by introducing a spacer between the chromophore and the nitroxide moiety [131]. A big advantage of photochemically triggered NMRP is the low reaction temperature at which the reaction can be performed. This not only minimizes the risk of side reactions but also allows for partial stereo-control which is not common in radical polymerization because of the planarity of the generated sp^2^ hybridized radicals. For this reason, polymerizations in the presence of additives and mediators or by using monomers with predetermined stereo specificity are commonly employed techniques to still gain control over stereospecifity [132,133,134]. Partial stereo-control, however, could also be achieved lately by photo NMRP where approximately 60% syndiotactic content was obtained [135]. 

An interesting feature of iniferter alkoxyamines is the possibility to fabricate macro photo-initiators. Chromophoric groups which are positioned on the nitroxide or alkoxy moiety can be transferred to the polymer and should be able to initiate a photopolymerization. In addition, this approach provides possibility to accomplish a dual initiation mechanism, where the NMRP is initiated thermally and the formed chromophore functionalized polymer is able to undergo photopolymerization in a second step [136]. The conceptually opposite way, which is the photochemical attachment of nitroxide moieties to a polymer backbone to yield a NMRP macroinitiator was demonstrated by our group. Benzophenone (BP) as *Norrish Type II* initiator in presence of TEMPO radicals was used to abstract hydrogen atoms of poly(ethylene oxide) (PEO) upon light irradiation. The TEMPO radicals can recombine with the formed radicals on the PEO backbone giving a TEMPO macroinitiator. Grafting from experiments demonstrated that TEMPO was attached successfully to the backbone and remains active for NMRP reactions [137]. The approach was further extended to visible light incorporation of TEMPO units to polyethylene (PE). PE-*g*-PSt copolymers were synthesized by combination of ring-opening metathesis polymerization (ROMP), Mn_2_(CO)_10_-Assisted TEMPO Substitution and NMRP [138].

One of the biggest advantages of NMRP is the accessibility of mono- or bi-functional polymers. This enables a facile approach to design complex polymer structures such as star polymers. A vast number of functional nitroxides are now reported in literature carrying e.g., chlorides, bromides, ketone, amine, alkoxy or ester groups on the alkoxide fragment (**N30**–**N35** in Chart 6) [139,140,141,142,143]. These compounds as well as other alkoxyamines such as TEMPO, TIPNO or SG1 [28,144,145,146,147,148,149,150] were used for the preparation of functional polymers [151,152,153] or to investigate the chain end fidelity of the polymers [142,145,154,155,156,157,158,159,160,161,162].

### 2.3. Application of NMRP in Materials Synthesis

The development of click chemistry inspired the synthesis of alkyne and azide functional alkoxyamines (**N30**, **N36** in Chart 6) [144,163,164] and synthesis of α-functionalized azide and alkyne polymers. Interestingly, polymerization with azide functional alkoxyamines appeared to be challenging and either click reaction had to be done before using the nitroxide as initiator (e.g., **N36** in Chart 6) or chloro functional alkoxyamines (e.g., **N32** in Chart 6) were used with subsequent substitution of the chloride to the azide group after polymerization. The first azide functional alkoxyamine capable of successfully initiating polymerization was reported by Braslau and co-workers. In this process, the azide functionality was conserved after NMRP. Specifically, the easiness to introduce groups capable of undergoing click reactions and the fact that NMRP is a transition metal free polymerization technique opened up new synthetic pathways in bio-conjugation applications. Besides alkyne-azide click reactions, thiol-ene Michael addition is a widely used technique for such applications. The design of a new alkene functionalized alkoxyamine based on the structure of the well-known SG1 nitroxide turned out to be a useful strategy for the synthesis of α-alkene and ω-nitroxide functionalized PSt. Interestingly, the alkene group on the alkoxy fragment was conserved during the polymerization reaction and was active in thiol-ene reactions afterwards [165].

Further examples where NMRP was employed in bio-conjugation made use of specially functionalized alkoxyamines which allow grafting to or grafting from reactions. Polymer peptide conjugates for example are an interesting class of biomaterials which show self-organizing properties [166,167,168] and the possible application as drug carriers or for molecular targeting [169,170,171]. They can be easily immobilized on surfaces with the polymer fragment to fabricate bioactive surfaces. Direct attachment of the peptide on the surface often causes structural changes of the peptide and can influence the activity of enzymes. Using a polymer spacer between the peptide and the surface is an easy way to overcome this problem [172].

A SG1 type *N*-hydroxysuccinimide (NHS) functionalized alkoxyamine was used to synthesize PMA comprising poly(ethylene glycol) (PEG) sidechains. The NHS functionalized polymer could then be completely or partially coupled to proteins such as lysozyme or a neuroprotective peptide [173]. The inverse sequence, which is first coupling the nitroxide to a peptide and the polymerization as grafting from the peptide afterwards, was elegantly demonstrated by Gigmes and co-workers. Thereby they could keep up the selectivity and obtained well defined conjugates [174,175]. In their approach a MMA–SG1 nitroxide attached to the glycine end-group of a preformed peptide *via* the carboxylate group was used as macroinitiator to obtain a St-protein conjugate [176]. Similarly, NMRP was used for both grafting to and grafting from approaches to essentially yield chitosan (CTS) modified polymers [177,178]. One of the first studies of CTS modified by NMRP reports the functionalization of *N*-phthaloylchitosan with 4-OH–TEMPO under γ-irradiation. The CTS-TEMPO macroinitiator was then used for grafting St [179] and SS [180]. Functionalization of the amino groups in PCTS with AAm or acrylate (Ac) fragments and radical addition of BlocBuilder^TM^ gave a macroinitiator for grafting St, MMA and AN [181].

Very recently Marque and co-workers reported the preparation of a series of interesting spin labelled alkoxyamines. A Finland-trityl radical, which gives an easily interpretable pattern in ESR spectroscopy, was chosen as spin label of a TEMPO type alkoxyamine. No significant changes in the dissociation constants between the spin labelled and the parent alkoxyamines were observed regardless of the position of the spin label in the structure. Furthermore, the radical centered on the bulky marker did not participate in the polymerization of St, leading to spin labelled PS. This enables many possibilities in the preparation of polymers for theranostics or in vivo imaging. Proposed applications are e.g., tracking the distribution of polymer drugs or to transfer magnetic properties to organic materials [182]. 

Multivalent alkoxyamines possessing more than one nitroxide fragment in the molecular structure are interesting initiators for the synthesis of star polymers and complex polymer structures. Early works concentrated on the ESR investigation of TEMPO biradicals, which are separated by an alkyl chain. It was found that the coupling between the two radicals was transmitted through space rather than through the alkyl bond. The coupling coefficient turned out to be larger for longer alkyl spacers and at higher temperatures, thus at conditions where the bending of the chain is more likely, facilitating radicals to approach each other [183]. For example, the dissociation rate of a TIPNO bisalkoxyamine, where the two TIPNO fragments are separated by a (–CH_2_–)_4_ chain was twice as big as that of the mono-alkoxyamine. The proposed interaction of the radicals through space [184] was confirmed shortly later by theoretical studies [185]. Increasing the spacer length, however, has a drastic effect on the performance in NMRP. The mobility of TEMPO bisalkoxyamines with different spacer lengths with (–CH_2_–)_1_ unit to (–CH_2_–)_4_ units decreases with increasing spacer length and leads to a partially uncontrolled behavior. This was observed in the case of the (–CH_2_–)_4_ spacer, which gave PSt with a trimodal GPC trace. A better control could be achieved in other cases where (–CH_2_–)_1_–(–CH_2_–)_3_ spacers were used [186,187,188]. Reduced mobility of the nitroxide often comes along with loss of control and is also observed for smaller bi-functional nitroxide initiators. Typically, polymerization proceeds in a controlled fashion at the beginning of the reaction, which leads to a two-arm macromolecule. At higher conversions, often one arm breaks and forms a dead chain. This behavior can be explained by the bimolecular process to form the dormant species. Thereby the macromolecule needs to be captured on both sides by a free nitroxide radical in order to keep the control of the polymerization which bears steric difficulties as the chains grow. Typically, the GPC traces show a bimodal distribution, which corresponds to the formation of one and two arm fractions [189]. Although control in NMRP with bi-functional alkoxyamines suffers from steric and mobility shortcomings, they are proved to be useful in the synthesis of complex block copolymers via “inside-out” and “outside-in” polymerization. For example, a PSt-*b*-poly(*t*-butylstyrene)(P*t*BS)-*b*-PSt triblock copolymer was prepared with a TEMPO based dialkoxyamine in a facile two step “*inside-out*” protocol. First, PSt was polymerized, giving a bisnitroxide core from which two PSt chains were grown. Subsequent initiation of *t*-butylstyrene (*t*BS) polymerization allowed inserting a P*t*BS block between the PSt blocks and the nitroxide fragment. The GPC trace of the first polymerization step showed a bimodal behavior, thus some dead chains were formed during the first step. In the second step both, the triblock polymer and alkyl terminated chains were formed [190]. Furthermore, the opposite approach of an “outside-in” polymerization was applied successfully in the preparation of poly(*t*-butyl acrylate)P*t*BA-*b*-PSt-*b*-P*t*BA and P*t*BA-*b*-PBA-*b*-P*t*BA triblock polymers in a two-step protocol. GPC trace for the PSt block showed again a bimodal distribution, while a unimodal distribution for the P*t*BA block was observed [191]. This approach was also extended to tri- and tetra-functional SG1 based initiators for the preparation of three and four arm star PSt with controlled polymer weights [192] or even to six- and twelve fold functional TIPNO based alkoxyamines for the formation of star polymers with St, MA, *N*,*N*-dimethylacrylamide (NNDMAA) and isoprene (I) [193]. Increasing the number of functional groups in the nitroxide, however, comes along with a steady loss of control during the polymerization which is mainly reflected in multimodal distribution of the GPC traces.

NMRP was used excessively in the preparation of block and graft copolymers. Monomer sequence for the preparation of block copolymers in NMRP is in general very flexible. However, unfavorable kinetic parameter or side reactions should be considered by the choice of the sequence. PBA-*b*-PSt e.g., can be prepared by first polymerizing BA and using the macroinitiator for the initiation of the PSt block. Typically, well defined block copolymers with low D are obtained. Changing the order, thus first polymerizing the PSt block and re-initiation of the BA block leads to a high D in most cases [28,194,195]. However, adding free nitroxide can overcome this problem and in this way, PSt-*b*-BA could be obtained in a well-defined fashion [196]. Similarly, the sequence must be well considered when PMMA block copolymers are prepared. 

## 3. Atom Transfer Radical Polymerization (ATRP)

**Scheme 4:**
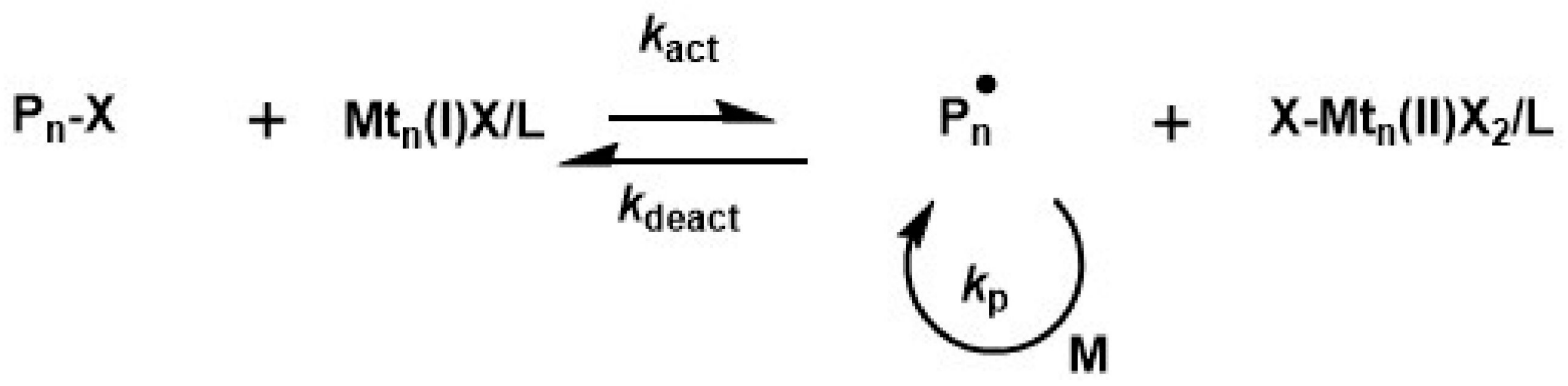
Activation and deactivation equilibrium reaction in ATRP.

ATRP is currently the most widely studied RDRP technique. Its appeal stems from the possibility to polymerize a wide range of monomers as well as the commercial availability of reagents and catalysts. In its pristine form, ATRP is catalyzed by transition metal (Mt_n_) catalysts such as Cu complexes with multi-dentate ligands. Control is achieved by establishing an equilibrium, in which a dormant species **R–X,** bearing a halide (typically alkylhalide or halide terminated polymer), is reversely activated by a Mt_n_ complex in its lower oxidation state. This activation step results in the formation of the Mt_n_ complex in its higher oxidation state and a radical capable to initiate polymerization or to propagate (see Scheme 4).

### 3.1. Mechanism in ATRP

The radical concentration **P_n_^∙^** is determined by the ATRP equilibrium constant (*K*_ATRP_) (see Equation (2)), the concentration of the alkylhalide and the ratio of the activator and deactivator concentration Cu(I)*X*/*L* and Cu(II)*X*_2_/*L*. Typical values of *K*_ATRP_ in Cu catalyzed systems [197,198] are below 10^−4^, thus the equilibrium is shifted to the left side and polymerization is typically operated at small radical concentrations to prevent termination.

(2) $$R_{P}=k_{p}[M][P_{n}^{*}]=k_{p}K_{\mathrm{ATRP}}(\frac{\left[P_{n}X][Cu(I)X/L][M\right]}{\left[X-\mathrm{Cu}(\mathrm{II})X_{2}/L\right]})$$

It is important to highlight that the ATRP rate constant, as it is given by Equation (2), depends on the ratio of activator and deactivator complex concentration. This means that in theory the amount of Cu species can be arbitrarily decreased, making Cu based ATRP a catalytic process. Cu mediated ATRP follows the PRE, thus each chain termination results in accumulation of the oxidized Cu species which eventually will drastically decrease *K*_ATRP_ as the reaction proceeds with time [199,200]. Mechanistic aspects of the catalytic cycle were thoroughly investigated. There is currently a debate about whether halogen transfer from **X–R** to the activator complex Cu(I)*X*/*L* proceeds through an inner-sphere electron transfer (ISET) process with a halogen bridging transition state [201,202,203,204,205,206] or through outer-sphere electron transfer (OSET). OSET can proceed in a stepwise mechanism (OSET-SW) under formation of the **RX^∙^** species or concerted (OSET-C) with electron transfer and bond cleavage happening in a single step. It was shown that electron transfer to an alkyl halide under ATRP conditions never gives **RX^∙^** and that OSET-SW can therefore be ruled out [203,205]. OSET-C, however, still remains a possible pathway in ATRP. The presence of metal and other transition metal catalysts introduces Cu impurities in the final product which are problematic regarding biological and electronical applications. In the past, different new ATRP techniques such as activators regenerated by electron transfer (ARGET) ATRP [207], initiators for continuous activator regeneration (ICAR) ATRP [208,209], electrochemically mediated (e) ATRP [210,211], or zerovalent metals or sulphite in supplemental activators and reducing agents (SARA) ATRP [212,213,214,215] were developed to achieve well controlled polymerization even at low catalyst loading. Inspired by photo-induced Cu alkyne azide click reactions [216] light triggered ATRP showed to be promising in minimizing the metal catalyst loading. Thereby, light induced ATRP not only proceeds faster with higher control at lower catalyst loadings but also allows for temporal and spatial control of the reaction [217,218,219,220,221,222,223]. 

### 3.2. Recent Developments in ATRP

**Scheme 5:**
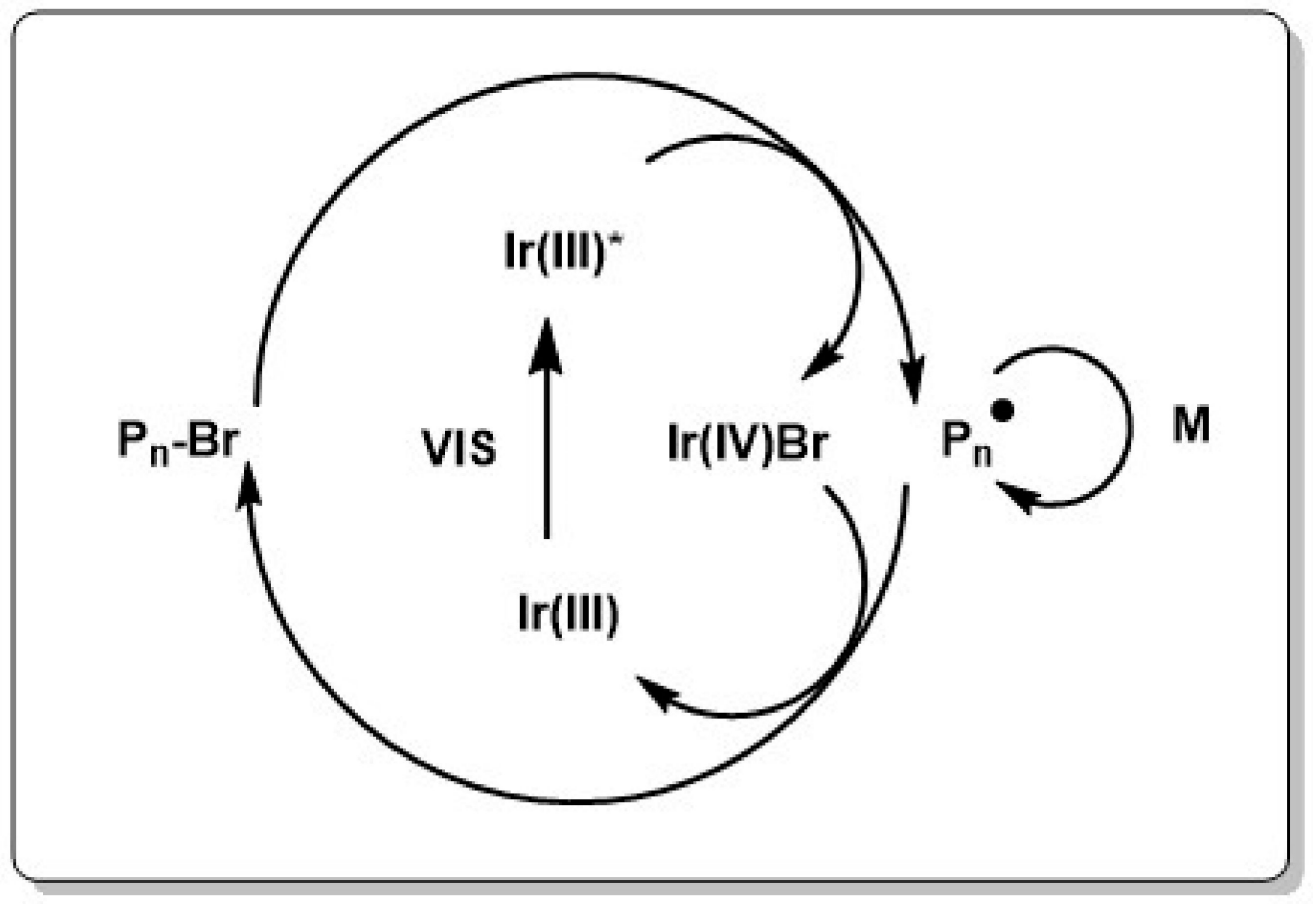
Schematic depiction of light catalyzed ATRP with Ir(ppy)_3_ as catalyst.

**Scheme 6:**
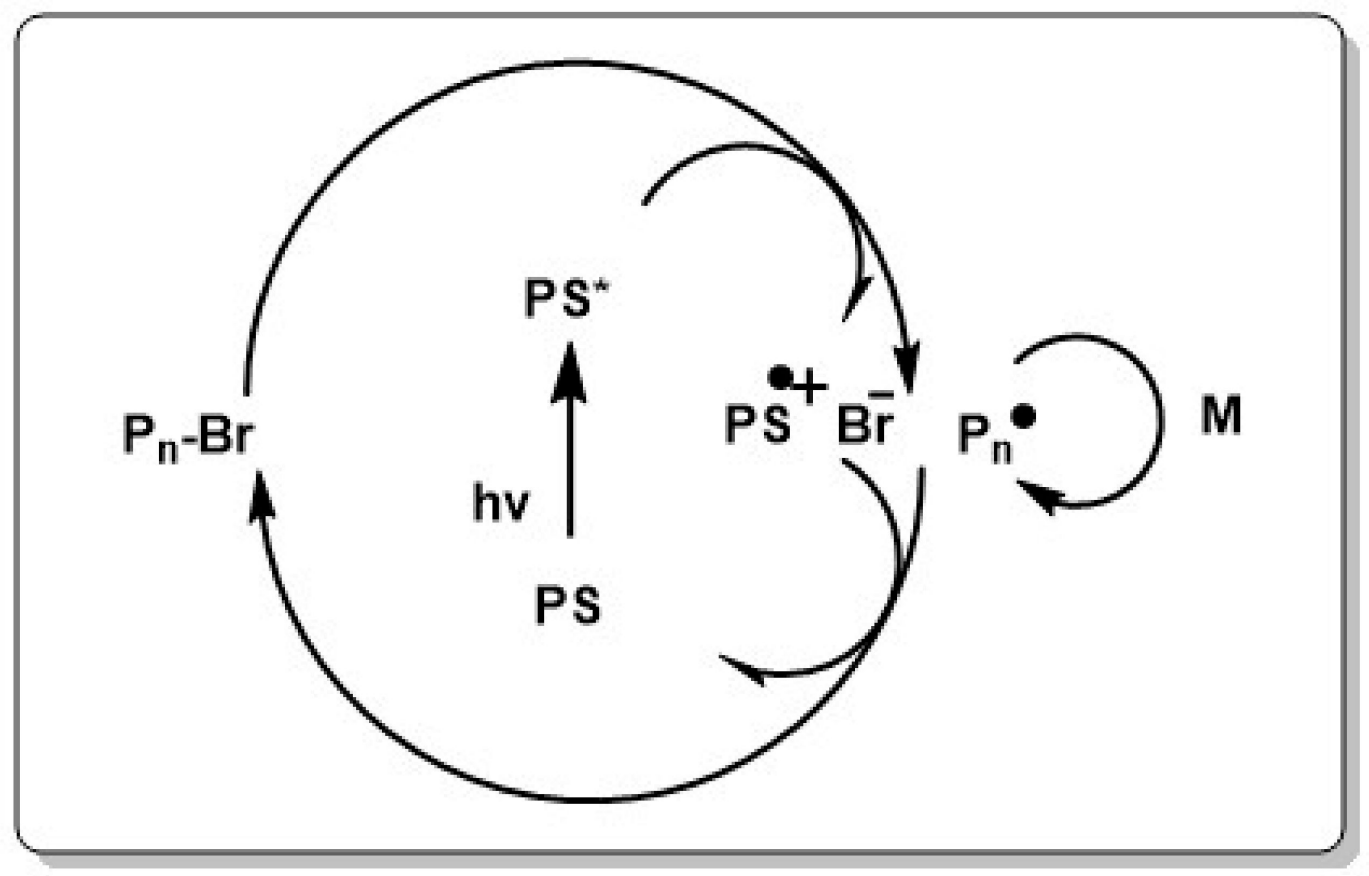
Schematic depiction of the oxidative quenching mechanism in metal free ATRP.

Mechanistically, photo ATRP can be activated through different light triggering reactions such as activation of a charge-transfer complex between the metal catalyst and the alkylhalide, activation of the inner sphere complex between catalyst and halide or generation of Cu(I)*X*/*L* from Cu(II)*X*_2_/*L* or vice versa. Recently our group could show that the spectral sensitivity can be extended to the visible spectrum by using commercially available photo-initiators [220,224,225], dyes [221], dimanganese decacarbonyl [226] or semiconductor photocatalysts [227,228], Lately in situ generation of Cu(I) from metallic Cu(0) without addition of photo-initiator was realized. Although this method showed dark polymerization, light strongly enhanced the polymerization rate. Additionally, using Cu(0) facilitates the handling of the catalyst and the recovery of the metal after polymerization [229]. An interesting case is the photo-induced ATRP polymerization with Ir(ppy)_3_ complex as catalyst. The *fac*-Ir(ppy)_3_ complex is able to reduce an alkylhalide upon irradiation to generate the Ir catalyst radical cation, bromine anion and an alkyl radical which is capable of initiating the polymerization. Deactivation and activation then proceeds as in conventional ATRP by switching between the Ir halide complex and the catalyst in its pristine form (see Scheme 5) [230]. ATRP reactions which follow this photocatalytic mechanism are commonly named as photo-induced electron transfer (PET) ATRP. Inspired by the photo-initiated oxidative quenching mechanism of Ir(ppy)_3_ it became soon clear that replacing the Ir catalyst with an organic catalyst, which is reducible in the excited state would lead to a stringent metal free system. To be able to replace the Ir catalyst, the organic catalyst must have a reduction potential in the excited state which is similar to the reduction potential of Ir(ppy)_3_ and must be able to form stable cation radicals which can coordinate reversibly a halide radical to act as deactivator throughout the polymerization (see Scheme 6).

Phenothiazine (**PTH**) has these characteristics and first attempts with 10-methylphenothiazine (**Me–PTH**) showed to be partially successful. While the experimental molecular weight agreed well with the theoretical molecular weight, the control over molecular weight distribution remained low. Tuning the nitrogen environment and replacing the methyl substituent with phenyl (10-phenylphenothiazine **Ph–PTH**) finally allowed for completely controlled polymerization, similar to conventional ATRP systems. It was assumed that change from the methylene to the phenyl substituent in the **PTH** reduces the catalyst decomposition, thus keeping the catalyst active for the deactivation step. This was later also supported by theoretical studies [231]. A detailed investigation of the metal free system showed that (i) the reaction is photo-catalyzed and no polymerization in absence of organocatalyst or light takes place and (ii) the reducing power of the excited state (*E*_red_(Ph–PTH^∙+^/Ph–PTH* = −2.1 V vs. SCE) is the driving force of the reaction. Replacing **Ph–PTH** with dyes which are oxidizing in the excited state such as Eosin Y and Methylene Blue, did not result in polymerization. The highly reducing excited state of **Ph–PTH** also allowed for a wider functional group tolerance. 2-(dimethylamine)ethyl methacrylate (MAEMI) which failed to polymerize under Ir(ppy)_3_ catalysis, could be polymerized with **Ph–PTH** [232]. Soon after the first report on the metal free system, different other dyes were investigated as catalysts in an oxidative quenching mechanism and subsequently the range of organocatalysts including benzo[b]phenothiazines [233], *N*-aryl and *N*,*N*-diarylphenothiazines and perylene [234,235], dihydrophenazine [231] and *N*-arylphenothiazines [234] acting under UV and visible light irradiation were successfully applied. The mechanism of the oxidative quenching is depicted in Scheme 6.

Our group lately compared the performance of pyrene and anthracene as potential catalysts for metal free ATRP. Results showed that at both low and high concentrations of anthracene polymerizations resulted in lower yields. Furthermore, the D turned out to be rather large and GPC traces showed a bimodal distribution when anthracene was used as catalyst. A detailed photochemical investigation revealed that excited anthracene can undergo either [4 + 4] cycloaddition with anthracene in the ground state, or reduces the alkylhalide to give alkyl radical, halide radical anion and the anthracene radical cation. The rate constant for the reduction step is rather high, which allows for efficient generation of radicals. However, the possibility of [4 + 4] cycloaddition is increased at higher concentrations, thus decreasing the polymer yield. Furthermore, NMR investigation revealed that polymer was grafted on the anthracene ring, which leads to the conclusion that anthracene radicals are not stable enough to prevent termination reactions via coupling. 

**Scheme 7:**
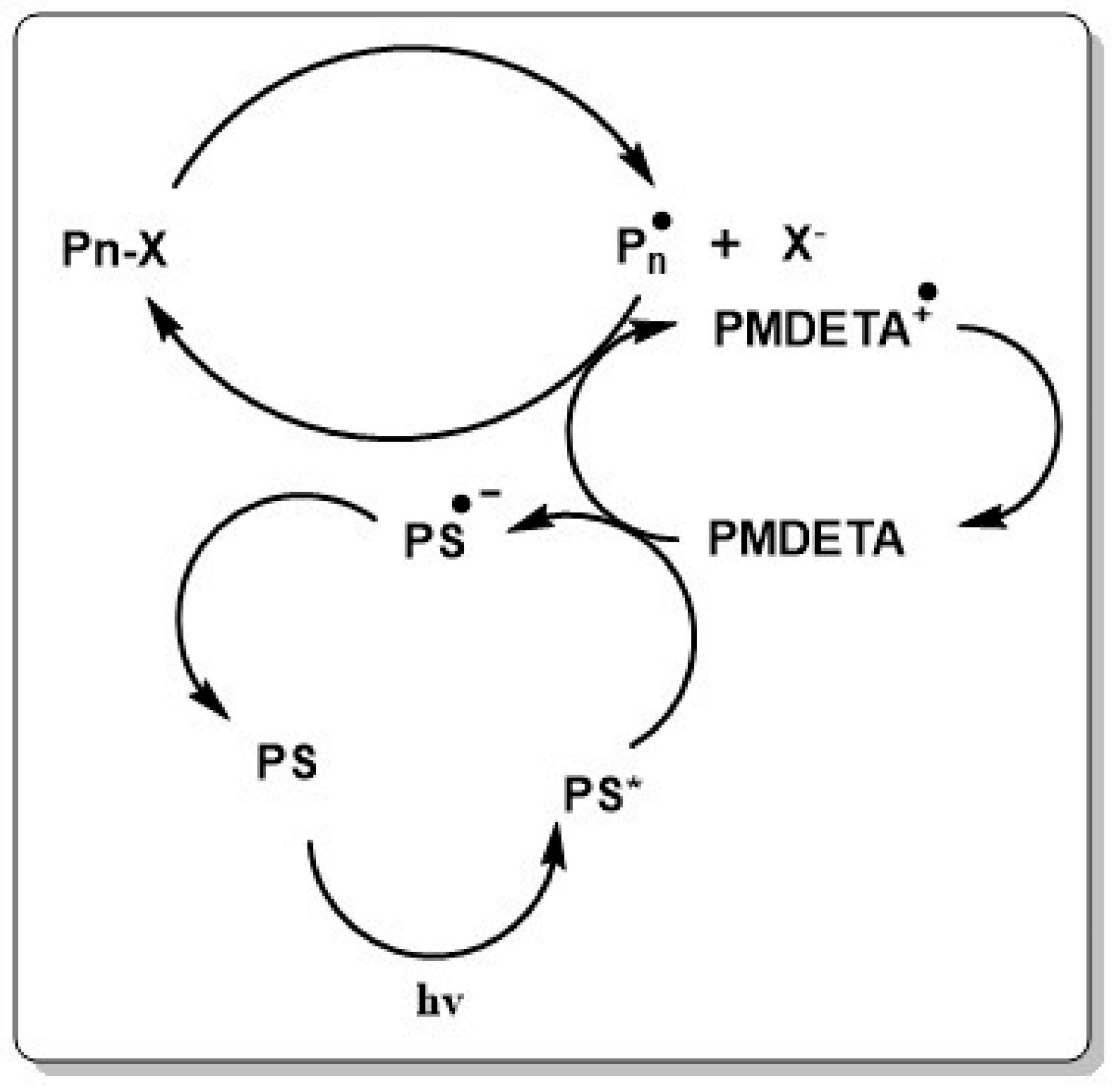
Reductive quenching mechanism with *N*,*N*,*N*′,*N*′′,*N*′′-pentamethyldiethylenetriamine (PMDETA) as an example for electron sacrificing compound in metal free ATRP.

In contrast, pyrene was much more tolerant towards the catalyst concentration. Initiation of the polymerization and the deactivation pathway followed the same way as anthracene but [4 + 4] cycloaddition did not occur. Excited pyrene, however, can form an exciplex with ground state pyrene molecules which can also undergo electron transfer with alkyl halides [236]. Very recently, we reported the successful photo-induced metal free ATRP using highly conjugated thienothiophene derivatives. Due to their high reducing nature these compounds favor an oxidative quenching mechanism [237]. Besides the oxidative quenching mechanism of excited state reducing dyes, the scope of metal free ATRP was lately extended to the reductive quenching mechanism (see Scheme 7) in presence of electron sacrificing compounds, such as amines. 

In the proposed mechanism, the excited state dyes undergo electron transfer with electron donor amines. The formed radical anion dyes reduce the initiator alkyl halide to yield radicals which are responsible for the initiation. A back electron transfer from the halide anion to the amine radical cation concludes the formation of the dormant macroalkyl halide which returns to the polymerization cycle [238]. A detailed review highlighting the mechanistic aspects of organocatalyzed ATRP was recently published [239]. We have also demonstrated that reductive quenching with commercially available photo-initiators can be used to control polymerization in a metal free ATRP system. BP, camphorquinone (CQ), thioxanthone (TX) and ITX as long wavelength photo-initiators were used in the presence of *N*,*N*,*N*′,*N*′′,*N*′′-pentamethyldiethylenetriamine (PMDETA) as electron sacrificing compound. Experiments showed all features of a RDRP and chain extension experiments were performed successfully [240]. Further studies included Eosin Y and Erythrosin B as reducible dyes which absorb in the visible spectral range [238,241] or Fluorescein [242], all showing good performance as catalysts in the reductive quenching mechanism. 

An initial problem in organocatalyzed photo ATRP was the necessity of a rather high catalyst loading which is typically in the range of 500–1000 ppm [231,232,233,234,235,236,237,238,239,240,241,242,243]. This not only introduces problems such as high cost but also runs against the demand for more sustainable “green” chemistry. Therefore, current research in metal free ATRP concentrates also on the development of highly efficient organocatalysts which are able to control polymerization even in the low ppm range. Very recently, the dicyanobenzene based donor-acceptor fluorophore 2,4,5,6-tetra(9H-carbazol-9-yl)isophthalonitrile (4CzIPN) displayed good performance when applied with only 15 ppm concentration, yielding 90% monomer conversion within 3 h [244]. Increasing the concentration of the catalyst resulted in higher D, which might stem from the tendency of 4CzIPN to independently initiate polymerization or the occurrence of side redox reactions during ATRP [245]. Another important parameter concerns light intensity as lower light intensity generates a lower amount of excited state catalyst and decreases the concentration of the catalytically effective species [246,247]. Although the oxidative quenching as well as the reductive quenching mechanism for dyes were investigated thoroughly and experimental results confirm the proposed mechanisms, the role of the excited state remained a matter of discussion. Regarding the activation and deactivation mechanism it was proposed that in metal free ATRP with **Ph–PTHZ** catalyst, the electron transfer activation step from the excited state of the catalyst to the alkylhalide most likely proceeds via OSET due to the strong negative reduction potential of the catalyst in the excited state. Regarding the deactivation step, it was proposed that the alkyl radical first oxidizes to a carbocation which subsequently traps a halide anion [248,249]. In polymerization of MMA and AN, the carbocation should be unstable and should lead to side reactions, which however, were not observed in the experiments. Recently, Matyjaszewski and co-workers reported a detailed mechanistic study on photomediated metal free ATRP using several **PTH** derivatives and other related compounds as photoredox catalysts [250]. The authors performed a detailed mechanistic study on metal free ATRP to identify the structure-reactivity relationship and to answer the question if electron transfer proceeds via ISET or OSET mechanism as well as to shed light on the deactivation mechanism. All employed catalysts possess a strong reduction potential in the excited singlet state, thus OSET might be the preferred pathway. Density functional theory (DFT) calculations revealed that electron transfer from the singlet excited state of the catalyst to the alkylhalide is a concerted dissociative electron transfer process (DET) which most likely involves a termolecular reaction of catalyst cation radical, alkyl radical and bromine anion. The most likely deactivation pathway was found to be an associative electron transfer from alkyl radical and halide anion to the catalyst radical cation to form the catalyst in its ground state and alkylhalide. In the presented calculations, it was assumed that the singlet excited state participates in the electron transfer process. This was, however, questioned lately, since also the triplet state possesses a reduction potential high enough to activate the reaction [251]. Experimental results from laser flash photolysis, fluorescence, phosphorescence and electron spin resonance spectroscopy revealed that **PTHs** in their triplet excited state can undergo electron transfer processes with alkyl halides in *N*,*N*-dimethylacetamide (DMA) solution. The development of photo-initiated ATRP covering the current status and future perspectives was recently summarized in an excellent review [252]. 

Besides using organic catalysts in metal free ATRP, a mechanistically surprisingly similar photo controlled technique called reversible complexation mediated polymerization (RCMP) evolved lately [253]. The development goes back to RCTP (see Scheme 1) which makes use of carbon centered radicals generated from alkyl iodides by organic catalysts [254,255,256,257]. Typically, the system consists of a conventional initiator, an alkyliodide as dormant species and O, Ge, Sn, P or N based catalysts [255]. Both techniques use iodine as capping agent and have the advantage of using nontoxic, cheap and easy to handle components. Both techniques, however, have important limitations with respect to monomer choice and molecular weight, thus limiting the system essentially to the polymerization of styrenics and methacrylates, leaving RDRP of acrylates unattainable [258]. In the proposed mechanism, the conventional initiator starts the polymerization reaction. The propagating polymer chain can then capture an iodide radical which stems from the iodine-catalyst complex (**A–I**) giving the dormant polymer chain **P–I** and **A^∙^**. The dormant species can then be activated subsequently by **A** to form the deactivator complex **AI^∙^**. The polymerization rate can be determined as

(3) $$R_{p}=R_{p,0}{\left(1+2(\frac{k_{t}^{\prime }}{k_{t}K})\frac{\left[AI\right]}{\left[P-X\right]}+(\frac{k_{t}^{\prime \prime }}{k_{t}K^{2}})\frac{{\left[AI\right]}^{2}}{{\left[P-X\right]}^{2}}\right)}^{-\frac{1}{2}}$$

where *R*_p,0_ is the polymerization rate without the deactivator catalyst **AI**. In RTCP, the polymerization rate decreases with increasing ration of (**AI**)/(**P–X**). While RCTP makes use of conventional initiators to start polymerization, RCMP works mechanistically different and resembles more the photo-induced metal free ATRP. The system is typically composed of an alkyliodide as initiator, an organic reducing amine such as tetrakis(dimethylamino)ethylene (TDAE) or triethylamine (TEA) as catalyst and the monomer. In the proposed mechanism, the amine abstracts an iodine radical from the dormant polymer chain to form an iodine-amine radical complex. This complex is unstable and reacts with a second iodine to form an iodine molecule amine complex. The propagating polymer chain can react with this complex to give the dormant species and the amine. In addition to TDAE and TEA, also tributylamine (TBA), *N*,*N*-dimethylethylenediamine (DMEDA), tetramethylethylenediamine (TMEDA), ethylenediamine (EDA) and NBS are used as amines, while 2-cyanopropyl iodide (CP–I) commonly serves as iodine source [259]. Lately RCMP could be extended to a wide range of different monomers such as BzMA, St, AN, 2-hydroxyethyl methacrylate (HEMA) and 2-(dimethylamino)ethyl methacrylate (DMAEMA).

### 3.3. ATRP in Materials Synthesis

The fast development of metal free ATRP led recently to first applications in surface polymerization from nanoparticles [260,261,262,263,264,265], development of metal free ATRP in continuous flow reactors [266] and application to polymerization of biomass based monomers [267]. 

The development of thermoplastic elastomers, which are derived from sustainable resources, gained recently attraction due to environmental issues which are raised by petroleum-based thermoplastic elastomers. Thermoplastic elastomers are typically ABA triblock copolymers in which A represents a glassy block and B a rubbery middle block. Polyacrylates, polymyrcene, polyesters or poly(menthide) are polymers which are synthesized from fatty acids or vegetable oil derived monomers. They show typically a low glass transition temperature (*T*_g_) and are therefore used as replacement of the rubbery middle-block in ABA triblock polymers. Lactide and lactone derivatives or rosin derivatives on the other side can be used as sustainable monomers in the synthesis of glassy end-blocks. Tang and co-workers reported lately the synthesis of well controlled ABA triblock copolymers comprising a PBA middle block and rosin-derived end-blocks by ATRP. By keeping the mid-block size constant but varying the end-block size, thermoplastic elastomers with elongation at break similar to petroleum derived elastomers and with block size dependent order-disorder temperatures were obtained [268]. 

Further examples of ATRP polymerization of biomass derived monomers led to the development of materials with interesting properties, such as shape memory polymers [269] or cellulose rosin copolymers with a tuneable *T*_g_ [270,271].

Very recently also photo-induced metal free ATRP was used for the polymerisation of soybean oil methacrylate (SBMA), furfuryl methacrylate (FMA) and dehydroabietic ethyl methacrylate (DAEMA). SBMA, which is derived from soybean oil, has long alkyl chains and can decrease the Tg in copolymers. Tang and co-workers used metal free ATRP with **PTH** as the photocatalyst to obtain polymers with low D and could show the synthesis of diblock copolymers with PSBMA as a soft block [268].

The development of organocatalyzed ATRP led also to application in biochemistry and biomedical sciences and in synthesis of polymer-protein conjugates. Lately, the immobilization of *N*-bromosuccinimide (NBS) initiator on proteins and ATRP polymerization with the protein initiator was reported [272]. A DNA synthesizer was used to prepare well-defined homopolymers, diblock copolymers and biohybrids by ATRP and MA as well as Ac polymers with excellent control were obtained. Grafting of hydrophilic and hydrophobic monomers from DNA was also reported [273]. The effective grafting under bio-relevant conditions of AAm, NNDMAA and *N*-vinylimidazole (NVI) from bovine serum albumin (BSA) as model protein via ICAR-ATRP was demonstrated lately. Both, homo and block copolymers could be grafted from the protein, thus livingness was retained during the polymerization [274]. 

## 4. Reversible Addition Fragmentation Chain Transfer Radical Polymerization (RAFT)

RAFT emerged as one of the most robust and versatile RDRP techniques since a first seminal publication by Rizzardo, Moad and Thang in 1998 [275]. Control of polymerization in RAFT is established by the addition of a chain transfer agent (CTA) or RAFT agent, typically a thiocarbonylthio compound (see Chart 1), which allows for reversible activation deactivation reactions. 

**Scheme 8:**
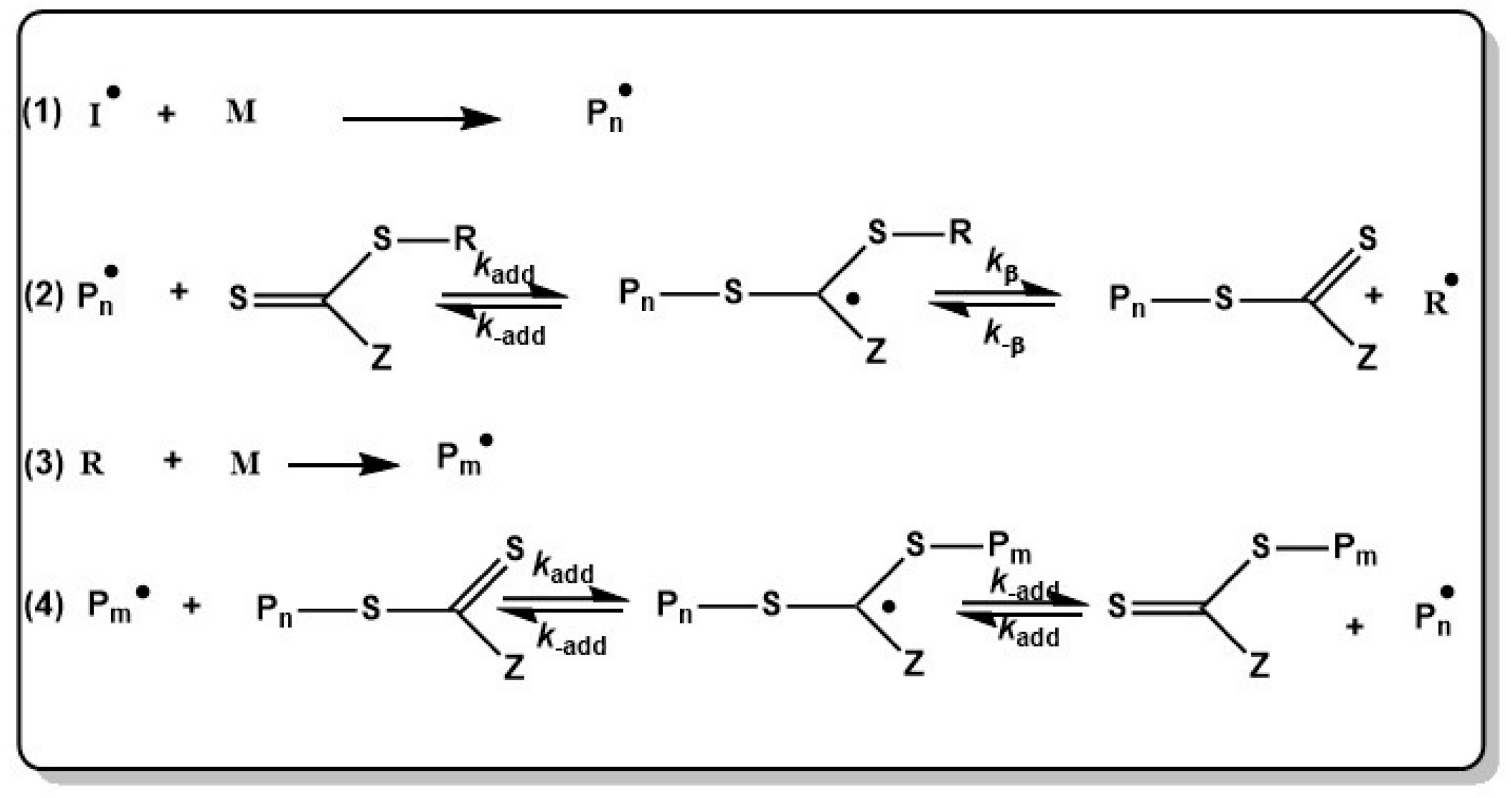
Mechanism of RAFT polymerization.

Importantly, the equilibrium between activation and deactivation reaction is established by DT (see Scheme 1), a process which involves only exchange of the functionality. Therefore, a conventional external initiator radical (**I^∙^**) is required in the initiation step (Equation (1) in Scheme 8). The oligomeric radicals (**P_n_^∙^**) formed at the first stage react with the CTA to give a radical intermediate. Fragmentation of the intermediate species releases a thiocarbonylthio group and a new radical (**R^∙^**). This newly formed radical can react with a thiocarbonyl group (back reaction in Equation (2) in Scheme 8) or initiate polymerization by reaction with monomer (Equation (3) in Scheme 8) and formation of a new propagating radical (**P_m_^∙^**). After the initialization period (Equations (1)–(3) in Scheme 8), which is the time in which the CTA is fully consumed, the equilibrium between active and dormant species is established (Equation (4) in Scheme 8).

### 4.1. Mechanism of RAFT Polymerization

The transfer constant (*k_tr_*) for the chain transfer can be defined in terms of rate constant for the addition (*k*_add_) and the fragmentation (*k*_β_) reaction as given in Equation (4).

(4) $$k_{\mathrm{tr}}=k_{\mathrm{add}}\frac{k_{\beta }}{k_{-\mathrm{add}}+k_{\beta }}$$

**Chart 7:**
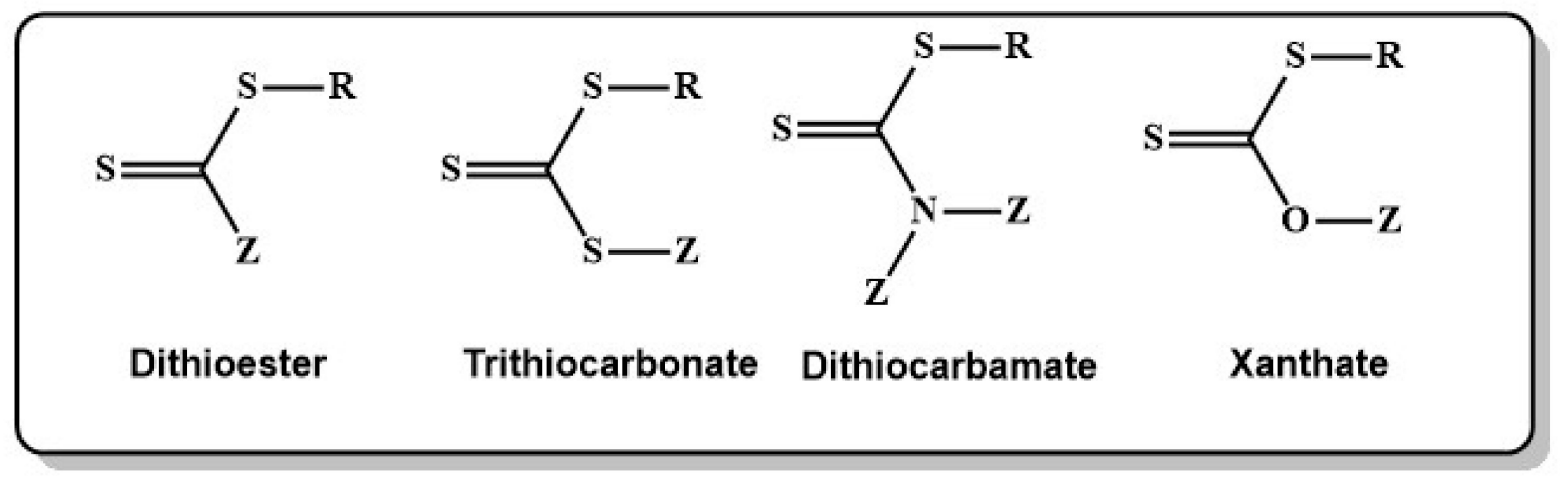
Typical chain transfer agents used in RAFT.

Transfer constants for different dithio compounds depend on Z, R (see Chart 7) and the type of monomer and can span more than five orders of magnitude, ranging from <0.01 to 1000. However, a transfer constant bigger than 2 is necessary to obtain small D [276,277]. A good RAFT agent should therefore possess a reactive C=S double bond (high *k*_add_), the intermediate radicals should have a high dissociation constant (high *k*_β_) and fragment in favor of the products (*k*_β_ ≥ *k*_add_) [278]. Bicciocchi et al. tried to decouple the R and Z group effects of the CTA and the monomer effect by polymerization of St with a benzylthiocarbonylthio RAFT agent with varying substitution of Z. Thus, changes in consumption of monomer and RAFT agent could be attributed to the influence of Z in the RAFT agent. The transfer constants decreased in the order Z = aryl > alkyl ~ alkylthio ~ pyrrole > aryloxy > amido > alkoxy > dialkylamino [279].

The mechanism proposed in Scheme 8 on the other side suggests that R must be a good homolytic leaving group relative to the propagating macromolecule as well as a radical capable of reinitiating polymerization. Importantly, the CTAs used as the RAFT agent act highly selective towards different types of monomers and must be carefully selected for each monomer system. More active monomers (MAM) such as MMA, St, MA and AN are polymerized under good control by dithioesters while the same CTA retard the polymerization of less activated monomers (LAM) such as vinylacetate (VAc), *N*-vinylpyrrolidone (NVP) and *N*-vinylcaprolactam (NVC). Latter ones, however, can be polymerized in a controlled way by xanthates (see Chart 7), *N*,*N*-dialkyl or *N*-alkyl-*N*-aryl dithiocarbamates. The equilibrium is typically rapidly established at the beginning of the polymerization, which guarantees, together with a fast initiation, a continuous growth of all polymer chains and yields polymers with narrow molecular weight distribution. Since conventional initiators are used in the initiation step, termination processes are unavoidable. The degree of termination reactions depends mainly on the initiator concentration and the dissociation rate of the initiator. Under typical RAFT conditions, the number of dormant thiocarbonylthio polymer chains is higher than the number of initiator derived chains and therefore most chains remain living. However, to maximize the chain fidelity, the initiator concentration can be minimized and a target molar mass, which is lower than that of polymer formed in absence of CTA (free radical polymerization condition), must be aimed for [278,280,281]. Additionally, the monomer conversion should be lower than 90% which makes isolation and purification of the polymer necessary before it can be used in chain extension reactions. 

According to the mechanism, the number of propagating species and the polymerization rate should not be affected by the concentration of the RAFT agent. However, the rate of polymerization in RAFT with dithiobenzoates as CTAs appears retarded with increasing CTA concentration [281] The retardation of the polymerization rate for some specific RAFT agents was intensively studied and three possible mechanisms are reported:

In the slow fragmentation model, retardation takes place due to a slow fragmentation of the intermediate two arms adduct. This results in a small fragmentation constant (large equilibrium constant), which is in agreement with the experimental observations. However, the model predicts concentrations of propagating radicals and intermediate adduct radicals which differ from the experimental conditions [281,282]. 

In the intermediate radical termination (IRT) model, the retardation is assumed to be caused by cross termination reaction between adduct radicals and propagating radicals [283]. The intermediate radical termination with oligomers (IRTO) model finally proposes termination between adduct radicals and oligomeric propagating radicals. The equilibrium constants and concentration of radical species are close to the experimentally obtained values. However, retardation should occur over the full conversion range which is not observed experimentally [284,285]. 

The partially contradictory models and the fact that a mechanism which holds in all cases could not be established, led to the formation of a IUPAC task group in 2005 with the aim to understand in detail the mechanism of RAFT polymerization. The group lately summarized the different models which partially explain the retardation in RAFT and an extended discussion in a report [286]. 

RAFT is the RDRP which comes closest to the conditions of conventional free radical polymerization. This is especially an advantage, since the strength of free radical polymerization, such as large choice of monomers, large range of solvents to be used [287,288,289], the possibility to carry out polymerization at a wide range of temperatures [290,291,292] and a high functional group tolerance [293,294] can be directly transferred to RAFT. RAFT essentially does not require high temperatures as for example NMRP or metal catalysts as in ATRP. This underpins its strength for biological applications [293]. Furthermore, RAFT appeared suitable for controlled polymerization of cyanoacrylates (CA) when macromolecular RAFT agents were used [295] while ATRP or NMRP are unsuitable for the polymerization of CA mainly due to the basic character of the reagents, such as amine ligands in ATRP. RAFT, however, also has some drawbacks, such as slow polymerization time, oxygen intolerance and the necessity to use external initiators, which increase the probability of termination reactions.

### 4.2. Recent Developments in RAFT

As outlined before, the choice of the right RAFT agent is crucial for successful polymerization. The efficiency highly depends on the interplay between the monomer and the properties of the leaving group R and the activity of the group Z [296]. The main families of RAFT agents are dithioesters [297], dithiocarbamates [298], trithiocarbonates [299] and dithiocarbonates (or xanthates) [298] (see Chart 7) with the common structure representing a Z group linked by a carbon, a nitrogen, a sulphur or an oxygen atom. Different phosphorus containing RAFT agents which bear the phosphorus atom in the R group, such as phosphine oxide-containing (R_1_R_2_P(O)–) R groups were developed and found to be useful for anchoring RAFT functionalities on the surface of inorganic quantum dots [300,301].

Furthermore, RAFT agents with phosphine and phosphine boron complex groups were applied for the preparation of end-group functionalized polymers as intermediates for the fabrication of polymer-peptide conjugates or biocompatible polymers [302]. First studies with agents bearing the phosphorus atom in the Z group were undertaken with phosphoryl and (thiophosphoryl)dithioformates of the formula R–S–(C=S)–P(X)(OR)_2_ (X = O or S) [303,304]. However, polymerization of St resulted in the formation of polymers with uncontrolled molecular weight, most probably due to a too slow rate of radical formation. Lately, Barner-Kowollik and co-workers were able polymerize 2-hydroxyethylacrylate (HEA) mediated by 2-cyano-prop-2-yl-(diethoxyphosphoryl)dithioformate [305] yielding polymers with small D. Furthermore, low molecular PSt could be obtained in a controlled polymerization by benzyl(diethoxyphosphoryl)dithioformate as the RAFT agent [306,307,308]. Dithiocarbamates can adopt zwitterionic forms which localize a positive charge on the nitrogen and a negative charge on sulphur atom. This reduces the double bond character of the thiocarbonyl unit and results in a lower reactivity towards radical addition and in smaller transfer constants [309,310]. As a consequence, they show reduced efficiency to polymerize MAMs [309]. The inhibition effect of dithioestes and trithiocarbonates on the polymerization of LAMs, on the other side, stems from the poor radical leaving group ability, which depends on the nature of R [311]. This selectivity effects hamper successful synthesis of block copolymers with MAMs and LAMs, such as the synthesis of poly(MAM)-*b*-poly(LAM) copolymers. To overcome this problem, stimuli-responsive RAFT agents which can polymerize both LAMs and MAMs were developed. *N*-(4-Pyridinyl)-*N*-methyldithiocarbamates for example can effectively polymerize LAMs such as VAc, NVP or NVC. In the presence of a strong acid, however, the protonated form can control successfully the polymerization of MAMs such as MA, BA and MMA [311,312,313]. Thus, switchable RAFT agents allow to synthesize block copolymers which are composed of more activated and less activated blocks, which would otherwise not be possible with typical RAFT agents [314]. For example PDMAA-*b*-PNVC block copolymer was prepared recently by the sequential polymerization of the respective monomers [315]. However, reactivity with respect to the different monomer classes is often compromised. Introducing electron-withdrawing groups to Z or R significantly enhances the transfer constant of the RAFT agents. This was lately exploited by the design of highly effective switchable RAFT agents with a pyrazole ring as Z group and substitution of the pyrazole in 4-position. The new agents appeared to be highly active in controlling the polymerization of MAMs and LAMs and PMAM-*b*-PLAM block copolymer formation [316].

**Scheme 9:**
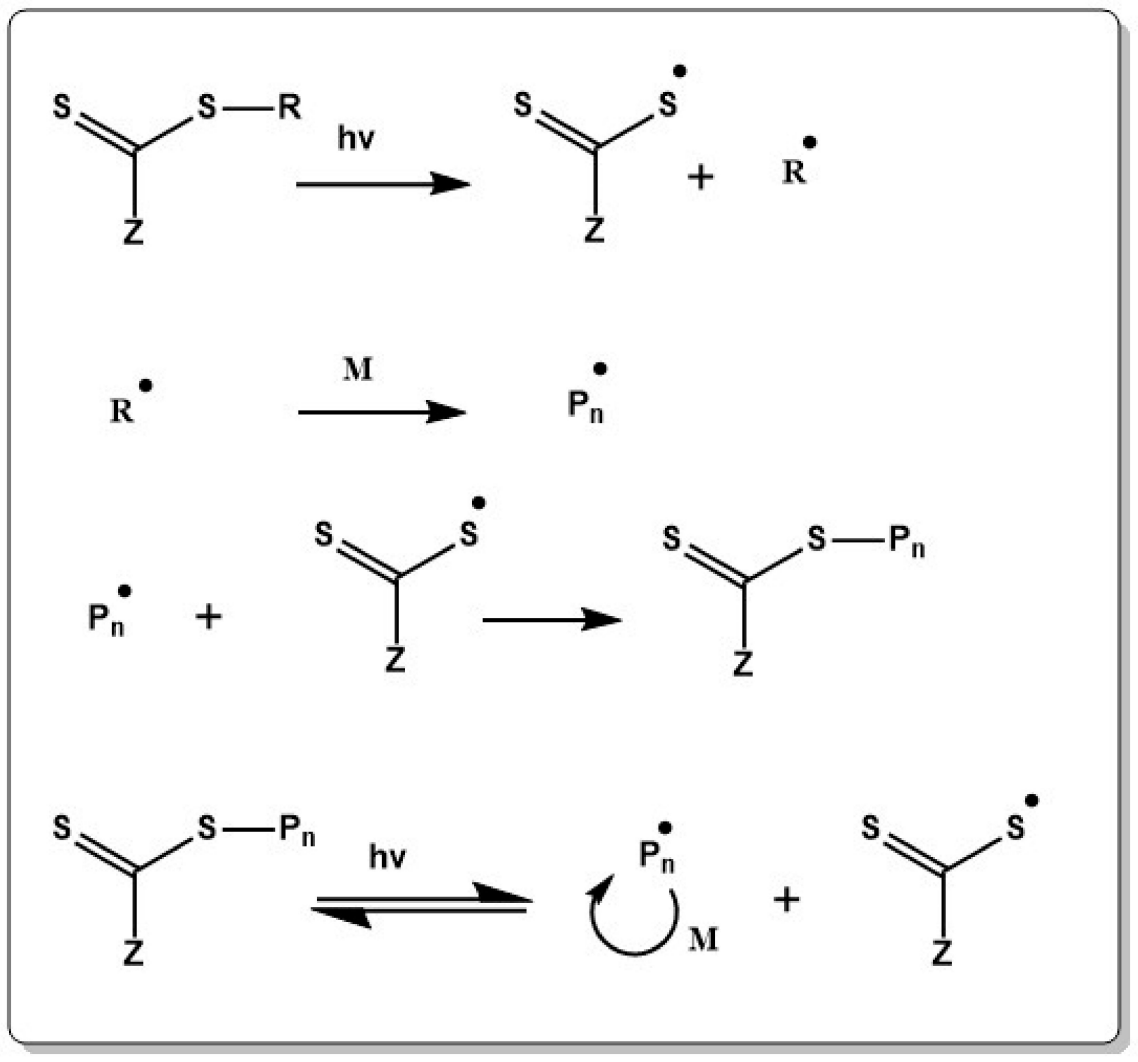
Reaction mechanism for photo-RAFT involving iniferter process.

Generally, thermal initiators are used in RAFT polymerization and the reaction is carried out at elevated temperatures. This causes problems associated with self-termination or irreversible chain transfer which lowers the degree of living chains [317,318,319]. Thus, extensive effort was put in finding alternative initiation methods, which allow performing RAFT under initiator free conditions or at lower temperatures. Obviously, initiation mechanisms which use light or high energy radiation appeared to be useful in developing RAFT polymerization free of external initiators. In the first report on irradiation initiated RAFT polymerization, MA, St and MMA was polymerized in the presence of dibenzyl trithiocarbonate under γ-irradiation resulting polymers with small D [320]. In the proposed mechanism, the S-benzyl linkage is sequentially cleaved under γ-irradiation liberating the benzyl radical as initiating species and a thiocarbonylthiyl radical. However, it soon became evident that the high energy radiation enables side reactions such as branching. Thus, initiating systems which respond to longer wavelength irradiation were developed. In the initial studies, dithiocarbamates termed as iniferters were used [321]. The reaction mechanism under UV light irradiation, however, most probably proceeds via a photochemical cleavage of the dithio compounds rather than through DT as depicted in Scheme 9 [322,323]. 

Polymerization of MMA and St under UV irradiation showed controlled behaviour and a narrow molecular weight distribution (MWD) at low monomer conversions. A significant broadening of the MWD was observed at higher monomer conversions, which was traced back to the decomposition of transfer sites at the end of the polymer chain and which eventually led to deactivation of the RAFT process [324]. The limitation in terms of conversion, however, can be overcome by using long wavelength photo initiators for radical generation and CTAs which do not absorb in the spectral regime of the radiation source. This was shown by Cai and co-workers who used S-dodecyl-S′-(α,α′-dimethyl-α′′-acetic acid) trithiocarbonate (DDMAT) and the long wavelength photo initiator (2,4,6-trimethylbenzoyl)diphenylphosphine oxide (TPO) as a bi-component system. Upon irradiation, the photo-initiator generates radicals which start the polymerization reaction and the RAFT polymerization proceeds according to the mechanism described in Scheme 8. Importantly, the wavelength of the light source must match the spectral response of the photo initiator but must not induce cleavage of the CTA. MMA could be polymerized in a controlled way with up to 85% conversion [325,326]. Furthermore, the choice of the photo initiator allows polymerization at long wavelengths, specifically at the visible range or under sunlight [327]. Very recently this approach was extended to RAFT polymerization of AN under blue LED light. 1,2,3,5-tetrakis(carbazol-9-yl)-4,6-dicyanobenzene was used as initiator in presence of 2-cyanoprop-2-yl-1-dithionaphthalate as transfer agent and gave PAN with narrow molecular weight distribution in a RDRP [328]. Inspired by a vast variety of phenacyl based photo initiators our group lately developed a phenacyl morpholine-4-dithiocarbamate (PMDC) RAFT agent for UV irradiated RAFT polymerization [329]. The phenacyl unit as R compound in PMDC appeared to be an easy leaving group, which is necessary for fast initiation. The polymerization of St proceeded in a controlled fashion and the morpholino thiocarbamate moiety was attached to the chain end with high end-group fidelity. Similar to the other reports on thiocarbonyl initiators [324,330] PMDC acted as RAFT agent in the process. Thus, light intensity as well as wavelength must be carefully selected to maintain high end-group fidelity in photo RAFT polymerization.

**Scheme 10:**
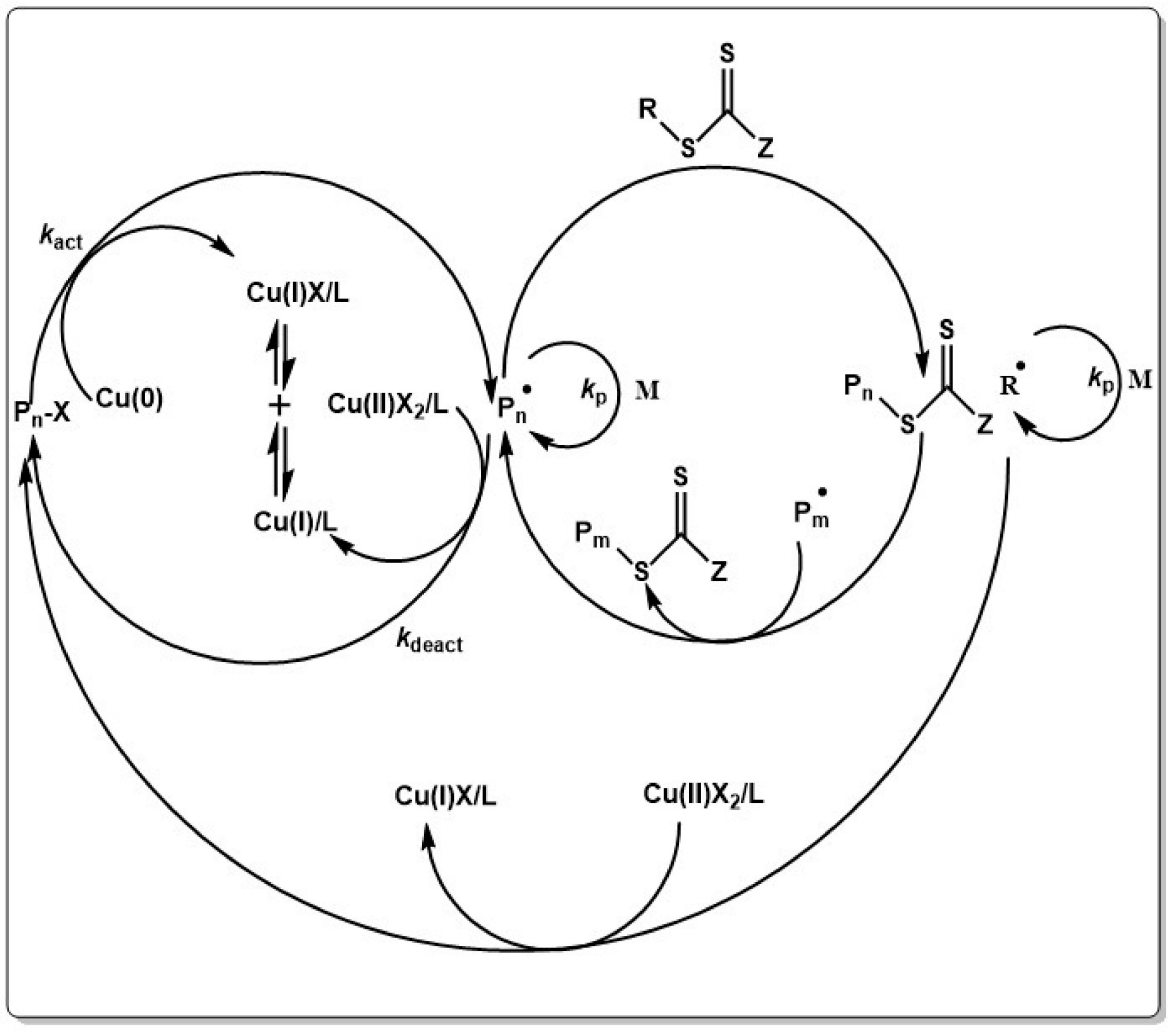
Reaction mechanism for PET-RAFT.

Sunlight activated RAFT polymerization by using a bifunctional thiocarbonylthio compound was realized, offering great promise for industrial applications. The reaction could be temporally controlled by light on/off experiments. Typically, thiocarbonylthio compounds show a distinct absorption in the visible region at 400–550 which stems from a *n* → π* transition [331]. By selective excitation with blue light at 460 nm, this transition allows for activation of a range of thiocarbonylthio compounds which can polymerize a vast amount of monomers [332]. A further step forward was the development of single electron transfer RAFT (SET-RAFT) which combines single electron transfer living radical polymerization (SET-LRP) via RAFT process. Typically, an alkyl halide oxidizes Cu(0) to yield an alkyl radical and Cu(I)X. Cu(I)X instantaneously disproportionates into Cu(0) and Cu(II)X_2_. The formed alkyl radical can react with the addition of monomer, or can be deactivated by the RAFT agent, releasing the R group of the RAFT agent as a radical along with the dormant species (see Scheme 10) [317,318,333,334]. 

Cu(0) species used in the original reports of SET-RAFT are sensitive to oxidizing environment and therefore, the reactions had to be carried out in a strictly inert atmosphere. Addition of reducing agents, such as hydrazine [335,336,337,338,339,340], strong acids [341] or ascorbic acid [342] allows to use insensitive CuO as catalyst and to carry out the reaction under ambient atmosphere. Application of SET-RAFT was extended to other transition metal catalyzed systems, such as iridium or ruthenium based catalysts [203,343,344], however, similar as in transition metal catalyzed ATRP traces of metals can potentially impede SET-RAFT for the application in biomedical or medical fields.

**Scheme 11:**
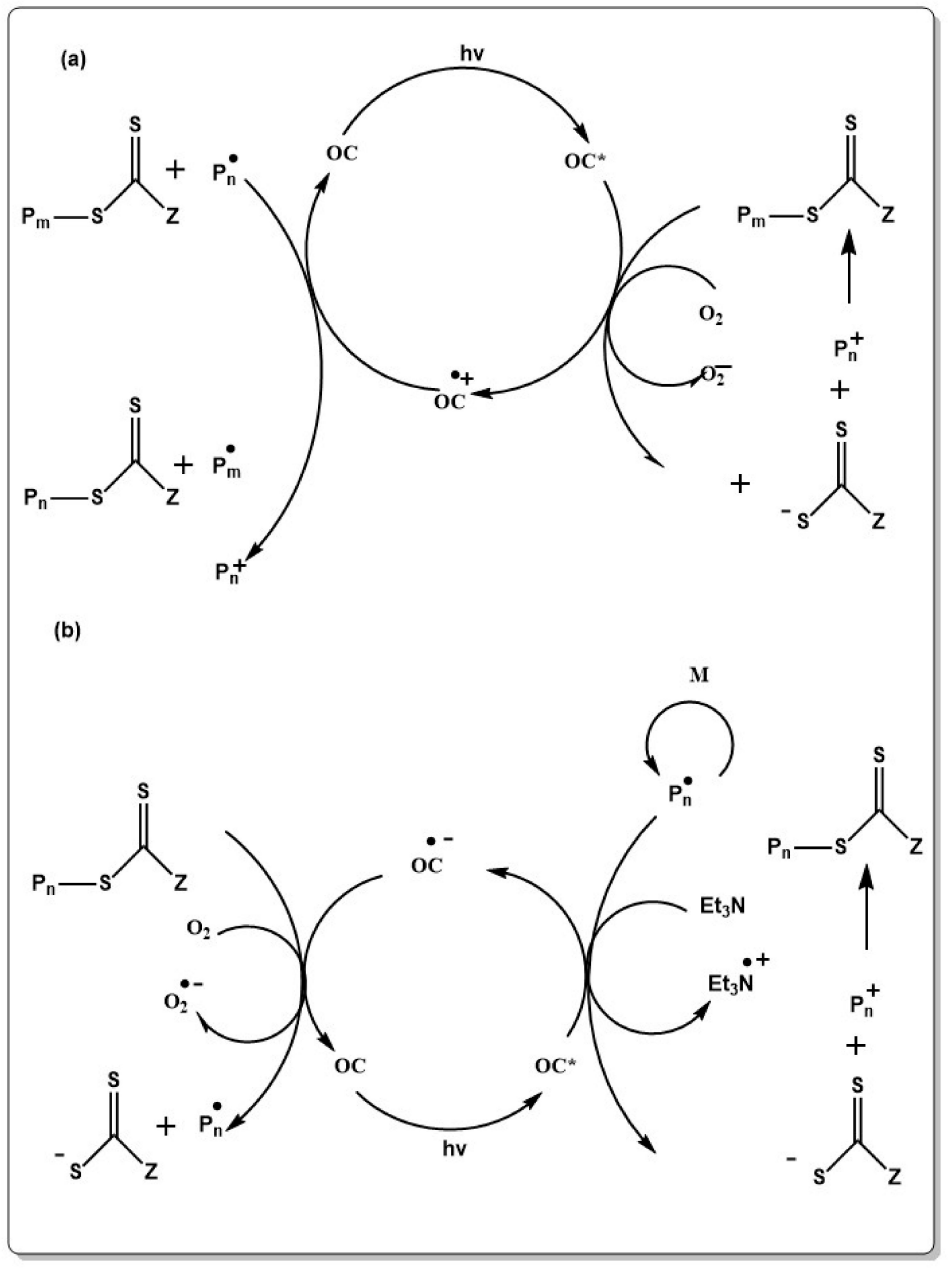
Organo catalyzed (OC) PET-RAFT. (**a**) Oxidative pathway; (**b**) reductive pathway.

Lately a transition metal free SET-RAFT process was developed in which sodium sulfites replace the conventional catalytic system. Inorganic sulfites were reported to activate dormant polymer chains during SARA-ATRP. Thus, application of these reducing agents in presence of 4-cyano-4-(phenylcarbonothioylthio)pentanoic acid as RAFT agent resulted in the polymerization of MMA. In the proposed mechanism, a reductive cleavage of the RAFT agent by one electron injection from the sulfite results in the formation of stable radical anions from the RAFT agent, which can then fragment in the initiating radical **R^.^** and the Ph–C(=S)–S anion. In accordance with the reaction involved in the process, the method is called dissociative electron transfer RAFT (DET–RAFT) [345]. The mechanism developed for PET–ATRP [230] was soon transferred to RAFT polymerization (PET–RAFT) [346]. The iridium complex *fac*-[Ir(ppy)_3_] was employed as photo catalyst and, upon irradiation, thiocarbonylthio compound was reduced via photo induced electron transfer. In the process, the thiocarbonylthio compound acts as initiator as well as CTA. The generated radical can participate in the RAFT reaction or regenerate the catalyst by reduction of the Ir(IV) species to Ir(III). The reaction can be performed at room temperature with catalyst loadings in the ppm range. This strategy was further extended to other catalytic systems, such as Ru(bpy)_3_Cl_2_ [346,347,348,349,350,351] and metalloporphyrins [352]. The aim to replace transition metal catalysts in RDRP led to the development of purely organic catalyzed photo RAFT systems. Boyer and co-workers investigated a series of organic dyes, namely Eosin-Y, Methylene Blue, Fluorescein, Nile Red and Rhodamine 6G as organocatalysts in the presence and absence of amine and with cyanopentanoic acid dithiobenzoate (CPADB) as CTA. Experiments revealed that the reactivity decreased in the order Eosin Y >> Fluorescein >> Nil Red, Rhodamine 6G and Methylene Blue. In the proposed mechanism, (see Scheme 11a) the organocatalyst in its excited state can transfer an electron to the RAFT agent. The RAFT agent acts as initiator and also as CTA to mediate chain growth in a living manner. Light on/off experiments with MMA showed that the photo polymerization remains dormant with no polymerization taking place in the dark state. 

Tertiary amines are known to be excellent reducing agents and capable to act as a sacrificial agent to provide an electron for the reduction of organocatalysts. Addition of TEA to Eosin Y catalyzed PET–RAFT resulted in doubling the polymerization rate with shorter induction periods. In presence of electron donors, a reductive mechanism (see Scheme 11b) is preferred over an oxidative pathway. In the reductive pathway, an electron transfer from the donor to the organocatalyst occurs. Under aerobic conditions part of the built catalyst radical anion will reduce the oxygen, while the other part will transfer an electron to the RAFT agent. Because of the mild conditions and oxygen tolerance of the reaction, PET–RAFT using organocatalysts was then further extended to aqueous systems which showed successful polymerization even in the presence of oxygen [353]. The process was also applied to biochemical systems, such as grafting from proteins [354].

Very recently the range of initiating systems was extended to enzymatic reactions. Considering the big amount of enzymatic catalytic systems in traditional radical polymerization and the mild reaction conditions used in RAFT, an enzyme catalyzed RAFT polymerization should be feasible. First attempts to use horseradish peroxidase (HRP) in RDRP, however, failed. Polymerization of poly(ethylene glycol) acrylate (PEGA) for example gave polymers with D larger than 1.5 [355] and control was completely lost upon polymerization of poly(ethylene glycol) methacrylate (PEGMA). Lately An and co-workers reported successfully a study on HRP initiated RAFT polymerization which showed control in polymerization of a wide range of MAMs and LAMs under different conditions, such as solution polymerization, dispersion polymerization and in biological environment [356]. The authors used the HRP/H_2_O_2_/Acetylacetone (AcAc) system, originally developed for enzyme catalyzed radical polymerization [357]. In the system, the enzyme undergoes a two electron oxidation which is driven by H_2_O_2_ to reduce two AcAc molecules. The AcAc radical species then initiates the polymerization. After the initiation step, the mechanism proceeds same as in a typical RAFT polymerization. Thus, enzymatic action is limited to the initiation step and does not participate in RAFT specific reaction steps. The reaction could be performed in a pH range from 4 to 7 and reached almost quantitative yield for NNDMAA with high chain end fidelity and narrow molecular weight distribution in short reaction times. The distinct pH dependence of the conversion was explained with the optimum reaction conditions of the enzyme as well as with the enol form of AcAc being the initiating species. Furthermore, an enzymatic cascade reaction using glucoseoxidase (GOx) and HRP could be demonstrated successfully. GOx catalyzes the oxidation of glucose by oxygen to H_2_O_2_ which then fuels the enzymatic reaction of HRP. Enzyme catalyzed RAFT was further extended to the synthesis of protein-polymer conjugates [358].

### 4.3. RAFT in Materials Synthesis

Since the development of RAFT, many multifunctional RAFT agents were reported in the literature and used for the preparation of star polymers for drug delivery [359,360,361]. Typically, two approaches are widely used in RAFT star polymerization. In the R-Star approach, the RAFT agent is attached via a reinitiating R group. In this method, however, linear by-products are commonly formed [362,363,364,365]. In the Z-Star RAFT polymerization the RAFT agent is attached via a stabilizing Z-group [366]. Homogeneous star polymers, however, can be best prepared by using a star RAFT agent that contains a stabilizing Z-group at the core [363,367,368,369]. Star polymer which are joint together by reinitiating R groups were employed in the fabrication of polymer gold nanocomposites. By joining the arms together though the R group, sulfur containing RAFT groups are displayed on the outer surface. After reduction, the sulfur groups can then attach to gold particles or surfaces [370,371,372,373]. This principle was employed by Rossner and co-workers to synthesize satellite nanostructures based on star polymers. The facile control of RAFT polymerization on the chain length thereby allows for tuning of the distance between the core in the middle and the satellites [374].

Since the RAFT mechanism proceeds via insertion of monomer into the C-S bond, end-functional polymers are obtained by using functional CTAs [311,375,376,377]. Incorporation of carboxylates [375], peptides [165], lipids [378], proteins [379] and many other groups [377,380,381,382,383] in the R group of the CTA for example led to end-functionalized polymers serving for specific applications. Obviously, the thiocarbonyl group is present on the ω-end of the polymer as a consequence of the polymerization mechanism. 

One of the prerequisites for successful RAFT is a weak C–S bond, which allows establishing the equilibrium during polymerization. However, the weak C–S bond can become a problem for practical applications. The thiocarbonyl fragment is easily released from the polymer and is responsible for colour appearance and odour. Thus, much effort was put in developing various methods for removing the thiocarbonyl end-group [380]. Furthermore, the thiocarbonyl group can be seen as protected thiol, alkene or dienophil which enables post polymerization reactions.

Sequence control during polymerization is becoming increasingly important in polymer chemistry. The first approach to achieve sequence control in RAFT goes back to Zard and co-workers, who reported RAFT single unit monomer insertion (SUMI) with xanthates as CTA for LAMs [384,385]. However, the reaction suffered from low yield and polymerization of activated monomers failed. Later on Moad [386,387] and Chen [388] could show that RAFT SUMI is achievable for MAMs when trithiocarbonate or dithiobenzoate was used as the RAFT agent. In general, a high transfer constant for the initial RAFT agent and a low rate constant for the propagation steps are beneficial. However, side product formation which makes purification by GPC necessary hampers the applicability is necessary. Initiator derived side reactions are one source of by-product formation. This problem could be solved by using light induced RAFT, where the RAFT agent is cleaved under UV light and initiates the polymerization as outlined before [389,390,391]. 

The robustness of RAFT and the possibility to carry out reaction in aqueous environment makes RAFT a suitable method for the synthesis of glycopolymers. Lately glycosylated core-shell colloidal nanoparticles were realised through surfactant-free emulsion copolymerization of sugars and St by RAFT. The reaction allowed obtaining core-shell particles with a narrow size distribution in a one-pot fashion. The size of the particles could be tuned by adjusting the sugar content with respect to the St content [392]. Grafting a glycopolymer from silica nanoparticle surfaces was realized lately by RAFT and binding of proteins to the glycopolymer was shown [393].

RAFT was also used intensively in the synthesis of block copolymers for drug delivery. Synthetic and neutral polymers showed great potential to improve bioavailability, shelf-life and therapeutic efficacy of drugs for oral administration. Spray drying is conveniently used to disperse active pharmaceutical compounds in polymers. Thereby polymer-drug interaction plays an important role in the performance of drug-polymer spray dried dispersions. Lately Reineke and co-workers investigated the effect of block chemistry and length towards polymer-drug spray dried formulations and solubility in oral delivery conditions. They used RAFT polymerization to synthesize five well defined block copolymers, namely poly(ethylene-alt-propylene), poly(*N*-isopropylacrylaminde) (PNIPAm) and poly(*N*,*N*-diethylaminoethyl methacrylate) (PDEAEMA), each copolymerized with a second block of a gradient copolymer of NNDMAA and 2-methacrylamidotrehalose. Thereby PNIPAm exhibits both hydrophilic and hydrophobic character depending on the temperature, while PDEAEMA being a hydrophilic and pH responsive polymer. They could show that the solubility of the polymer matrices in the dissolution media and an increase in hydrogen bonding sites in the polymer matrix are critical to decrease the drug crystallization, increase the drug solubility and achieve supersaturation concentration of hydrophobic drugs in the dissolution media [394,395].

Block polymers also find use as toughening agents, thermoplastic elastomers and pressure sensitive adhesives. For example, traditionally PSt-PI-PSt triblock copolymers are used as pressure sensitive adhesives. Lately, however, it was shown that acrylic block copolymers showed certain advances over St based polymers mainly in terms of UV light and oxidation stability. Furthermore, the functional versatility of acrylates allows forming block polymers with a broad range of polarity and *T*_g_ values. Additional the combination of biobased resources with petroleum based feedstocks became a vital aspect to improve the sustainability of pressure sensitive adhesives. Isosorbide, a bicyclic sugar derivative gained much attention as biobased feedstock lately because it imparts desireable qualities such as high *T*_g_ and thermal stability. Recently polymerization of isosorbide monomers was extended to RDRP because of the good control over the polymerization reaction and narrow D [396,397,398]. Furthermore, glucose based triblock copolymers were prepared by RAFT, where Glucose-6-acrylate-1,2,3,4-tetraacetate was used as the glassy component in thermoplastic elastomers [399]. 

## 5. Conclusions and Outlook

Significant advances in RDRP provided the basis to control the composition of polymers on a microscale and provides now the ability to fabricate materials which were not accessible a decade ago. Since the first reports on living anionic polymerization until the transfer of the living polymerization concept to radical polymerization, the synthetic techniques to precisely determine the structure of polymers were steadily improved. Today, we have a wide choice of methods to synthesize living polymers in a controlled fashion out of almost an arbitrary choice of monomers and terminated by a wide range of chemical functions. This enables to build up more and more complex structures and to develop tailored polymer materials for very specialized applications.

Research work during the last decade provided deep insight in the mechanisms governing control in radical polymerization. This allowed for developing new and highly efficient initiation or mediating systems which are able to provide a high degree of control during the reaction. Development of photo-induced RDRP in NMRP allowed to reduce reaction temperatures and to decrease the amount of side reactions. Photo controlled catalytic systems also allowed to minimize the amount of transition metal catalysts in ATRP or even to develop purely organic and metal free ATRP systems. Switchable RAFT agents allow for polymerization of more or less activated polymers, whose limitation was one of the big drawbacks in RAFT for long time.

The possibility to carry out RDRP under very mild conditions resulted in increased research effort in using these techniques for the synthesis of biocomposites, polymer-protein conjugates, glycopolymers or polymers for biomedical applications. Furthermore, RDRP finds more and more application in developing high end materials from renewable biopolymers. The full potential of the technique in these emerging fields, however, will just be explored in the future.

## Abbreviations

4CzIPN	2,4,5,6-tetra(9H-carbazol-9-yl) isophthalonitril
4VBC	9-(4-vinyl-benzyl)-9H-carbazole
Ac	Acrylate
AAm	Acrylamide
AcAc	Acetylacetone
AIBN	2,2′-azobis(2-methylpropionitril)
AN	Acrylonitrile
ARGET ATRP	Activator Regenerated by Electron Transfer ATRP
ATRP	Atom Transfer Radical Polymerization
BA	Butyl acrylate
tBA	*t*-butyl acrylate
BAI	Bis-(alkylphenyl)iodonium hexafluorophosphate
BMI	*N*-benzylmaleimide
BP	Benzophenon
BPO	Benzoylperoxide
tBS	*t*-butylstyrene
BSA	Bovine Serum A
BzMA	Benzyl methacrylate
CA	Cyanoacrylate
CMI	*N*-cyclohexylmaleimide
CPADB	Cyanopentanoic acid dithiobenzoate
CP–I	2-cyanopropyl iodide
CQ	Camphorquinone
CTA	Chain Transfer Agent
CTS	Chitosan
D	Dispersity
DAEMA	Dehydroabietic ethyl methacrylate
DBN	Di-*t*-butyl nitroxide
DC	Dissociation-Combination
DCF	Dead Chain Fraction
DDMAT	S-dodecyl-S′-(α,α′-dimethyl-α′′-acetic acid) trithiocarbonate
DET	Dissociative Electron Transfer
DET-RAFT	Dissociative Electron Transfer RAFT
DFT	Density Functional Theory
DMA	*N*,*N*-dimethylacetamide
DMAEMA	2-(dimethylamino)ethyl methacrylate
DMEDA	*N*,*N*-dimethylethylenediamine
DMPA	2,2-dimethoxy-2-phenylacetophenone
DPAIO	2,2-diphenyl-3-phenylimino-2,3-dihydroindol-1-yloxyl
DT	Degenerative Exchange Chain Transfer
e-ATRP	Electro ATRP
EDA	Ethylenediamine
ESR	Electron Spin Resonance
FMA	Furfuryl methacrylate
GOx	Glucose Oxidase
HEA	2-hydroxyethyl acrylate
HEMA	2-hydroxyethyl methacrylate
HRP	Horseraddish Peroxidase
I	Isoprene
ICAR ATRP	Initiators for Continuous Activator Regeneration ATRP
IRT	Intermediate Radical Termination
IRTO	Intermediate Radical Termination with Oligomers
ISET	Inner Sphere Electron Transfer
ITX	Isopropyl thioxanthone
LAM	Less Activated Monomers
MA	Methacrylate
MAEMI	2-(dimethylamine)ethyl methacrylate
MAM	More Activated Monomers
Me-PTH	10-methyl phenothiazine
MMA	Methyl methacrylate
MTEMPO	4-methoxy TEMPO
Mt_n_	Transition Metal
MWD	Molecular Weight Distribution
NBS	*N*-bromosuccinimide
NHS	*N*-hydroxysuccinimide
NMMA	Methyl-2-methyl-3-nitro-2-nitrosopropionate
NMRP	Nitroxide Mediated Radical Polymerization
NNDMAA	*N*,*N*-dimethylacrylamide
NPI	Maleimide-*N*-phenylmaleimide
MMA	*N*-vinylcaprolactam
NVI	*N*-vinylimidazole
NVP	*N*-vinylpyrrolidone
OSET	Outer Sphere Electron Transfer
OSET-C	Continuous Outer Sphere Electron Transfer
OSET-SW	Stepwise Outer Sphere Electron Transfer
PDEAEMA	poly(*N*,*N*-diethylaminoethyl methacrylate)
PEG	Poly(ethylene glycol)
PEGA	Poly(ethylene glycol) methyl ether acrylate
PEGMA	Poly(ethylene glycol) methyl ether methacrylate
PEO	Poly(ethylene oxide)
PET-ATRP	Photo-induced Electron Transfer ATRP
PET-RAFT	Photo-induced Electron Transfer RAFT
Ph-PTH	10-phenyl phenothiazine
PMDC	Morpholine-4-dithiocarbamate
PMDETA	*N*,*N*,*N*′,*N*″,*N*″-pentamethyldiethylenetriamine
PNIPAm	Poly(*N*-isopropylacrylamide)
PRE	Persistent Radical Effect
PTH	Phenothiazine
RAFT	Reversible Addition Fragmentation Chain Transfer Polymerization
RCMP	Reversible Complexation Mediated Polymerization
RCT	Reversible Chain Transfer
RCTP	Reversible Chain Transfer Polymerization
RDRP	Reversible-Deactivation Radical Polymerization
ROMP	Ring-Opening Metathesis Polymerization
SARA-ATRP	Supplementary Activator and Reducing Agent ATRP
SBMA	Soybean oil methacrylate
SET-LRP	Single Electron Transfer Living Radical Polymerization
SET-RAFT	Single Electron Transfer RAFT
SG1	*N*-*t*-butyl-1-diethoxyphosphoryl-*N*-oxidanyl-2,2-dimethylpropan-1-amine
SS	Styrene sulfonate
St	Styrene
SUMI	Single Unit Monomer Insertion
TBA	Tributylamine
TDAE	Tetrakis (dimethylamino) ethylene
TEA	Triethylamine
TEDIO	1,1,3,3,-tetraethylisoindolin-2-oxyl
TEMPO	2,2,6,6,-tetramethyl-2-piperidin-1-oxyl
TIPNO	2,2,5-trimethyl-4-phenyl-3-azahexane-3-nitroxide
TMEDA	Tetramethylethylenediamine
TPO	2,4,6-trimethylbenzoyl-diphenylphosphine oxide
TX	Thioxanthone
V70	2,2′-azobis(4-methoxy-2,4-dimethyl valeronitrile)
Vac	Vinyl acetate
